# Pediatric dentistry systematic reviews using the GRADE approach: methodological study

**DOI:** 10.1186/s12903-024-04542-w

**Published:** 2024-07-13

**Authors:** Rachel Alvarenga-Brant, Sarah Queiroz Notaro, Cristine Miron Stefani, Graziela De Luca Canto, Alexandre Godinho Pereira, Luciana Póvoa-Santos, Ana Clara Souza-Oliveira, Julya Ribeiro Campos, Carolina Castro Martins-Pfeifer

**Affiliations:** 1 Department of Surgery, Clinical Dentistry and Oral Pathology and Oral Surgery, Dental School, Federal University of Minas Gerais, Belo Horizonte, Brazil; 2 Department of Pediatric Dentistry, Dental School, Federal University of Minas Gerais, Belo Horizonte, Brazil; 3 Department of Dentistry, University of Brasília, Brasília, DF Brazil; 4 Department of Dentistry, Federal University of Santa Catarina, Florianópolis, Brazil

**Keywords:** Clinical trial, Pediatric dentistry, Child, Oral health, Dental care, Overview, GRADE approach, Systematic review, Meta-analysis

## Abstract

**Background:**

To assess the reporting of the certainty of the evidence using the GRADE approach in systematic reviews of interventions in pediatric dentistry.

**Methods:**

The inclusion criteria were systematic reviews of randomized clinical trials (RCTs) and non-randomized studies of interventions (NRSIs) in pediatric dentistry that reported the certainty of the evidence through the GRADE approach. Paired independent reviewers screened the studies, extracted data, and appraised the methodological quality using the Assessing the Methodological Quality of Systematic Reviews (AMSTAR 2) tool. The certainty of the evidence was extracted for each outcome. A descriptive analysis was conducted.

**Results:**

Around 28% of pediatric dentistry reviews of interventions used the GRADE approach (*n* = 24). Twenty reviews reported 112 evidence outcomes from RCTs and 13 from NRSIs using GRADE evidence profile tables. The methodological quality was high (16.7%), moderate (12.5%), low (37.5%), and critically low (33.3%), fulfilling the majority of the AMSTAR 2 criteria. The certainty of the evidence for outcomes generated from RCTs and NRSIs was very low (40.2% and 84.6%), low (33.1% and 7.7%), moderate (17.8% and 7.7%), and high (9.8% and 0.0%). The main reasons to downgrade the certainty were due to (for RCTs and NRSIs, respectively): risk of bias (68.8% and 84.6%), imprecision (67.8% and 100.0%), inconsistency (18.8% and 23.1%), indirectness (17.8% and 0.0%), and publication bias (7.1% and 0.0%).

**Conclusion:**

The proportion of systematic reviews assessing the certainty of the evidence using the GRADE approach was considered small, considering the total initial number of published pediatric dentistry reviews of intervention. The certainty of the evidence was mainly very low and low, and the main problems for downgrading the certainty of evidence were due to risk of bias and imprecision.

**Registration:**

PROSPERO database #CRD42022365443.

**Supplementary Information:**

The online version contains supplementary material available at 10.1186/s12903-024-04542-w.

## Background

Evidence-based dentistry has contributed substantially to improve oral health quality in general and the pediatric population more specifically. A systematic survey published in 2014 presented high-quality Cochrane systematic reviews (SRs) in pediatric dentistry [1]. However, that review did not evaluate the certainty of evidence, which has only more recently been analyzed methodologically in medicine and dentistry [2, 3]. In the medical field, only 43.6% of Cochrane reviews assessed the GRADE approach (Grading of Recommendations, Assessment, Development, and Evaluation) using a summary of findings (SoF) Table [3]. In dentistry, Cochrane reviews were 12 times more likely to assess the certainty of evidence in comparison to non-Cochrane reviews [2]. Recent methodological surveys in dentistry found mainly low and critically low-quality SRs [4–6]. Among them, one survey found that 54.3% of SRs in orthodontics assessed the certainty of the evidence through the GRADE approach, and 34.8% of those studies correctly followed all GRADE criteria [4]. In periodontology, 25.2% of SRs included the GRADE approach, and 37.5% of those studies followed all the GRADE criteria [5].

A systematic and explicit approach of study evidence can help prevent errors, facilitate critical assessment, and improve information dissemination. The GRADE approach enables more consistent analysis of the evidence, and subsequent communication can support better-informed choices in health care [7].

By assessing the methodological quality of SRs and the certainty of evidence, it is possible to identify gaps for improvement in these studies. A methodological survey published in 2015 found that most of the evidence in pediatric dentistry was low or very low [8]. Only 15 SRs on fluorides and caries prevention reported high to moderate evidence of 81 SRs evaluated. By searching for gaps through methodological surveys, we can identify which areas of pediatric dentistry need more research and which have provided high-quality evidence, resulting in an efficient use of time, money, and research effort. Therefore, this methodological survey aims to evaluate the certainty of evidence using the GRADE approach in pediatric dentistry.

## Methods

### Eligibility criteria

This methodological study included SRs and meta-analyses of interventions in pediatric dentistry published from January 1st, 2020 to March 3rd, 2022. All studies published during this period were considered for inclusion, characterizing a convenience sample. The study followed the Preferred Reporting Items for Systematic Reviews and Meta-Analyses (PRISMA 2020) [9].

To be considered a SR, the following criteria had to be fulfilled [6, 10]: clear clinical question with the PICO acronym (patient, intervention, comparison, outcome), clear systematic search, reproducible methodology, assessment of risk of bias, systematic presentation and synthesis of results. For inclusion, SRs should have included randomized controlled trials (RCTs) or non-randomized studies of interventions (NRSIs) that reported the certainty of the evidence using the GRADE approach. Included original studies had to be related to pediatric treatments in babies, children, or adolescents. There was no limitation regarding the language of publication.

Exclusion criteria: reviews including original studies with individuals older than 18 years, treatments or outcomes in other fields (e.g., orthodontic, endodontics in permanent teeth, surgery); other types of SRs/meta-analyses (observational studies of exposures related to the outcome (PECO), of in vitro/animal studies, and others), guidelines, scoping reviews, overviews (reviews of reviews), narrative reviews, letters/editorials of SRs, letters to the editor, cases/case series, other original studies (such as in vitro, animal studies, observational and clinical studies), SRs not reporting the GRADE approach and those using different approaches for assessment of the level or quality of evidence, review protocols, network meta-analyses.

### Information source and search strategy

Five electronic databases were searched: MedLine, Embase (both through Ovid), Cochrane Database of Systematic Reviews, Scopus and Web of Science. Supplementary Table S1 shows detailed search strategies for each database. Subsequently, the studies were organized in EndNote software, version X9.3.1 (Philadelphia, PA: Clarivate Analytics) for the removal of duplicates.

### Study selection

Independent paired reviewers screened titles and abstracts (SQN/JRC and ACSO/LPS) based on the predefined eligibility criteria using the Rayyan QCRI website. The same reviewers independently screened the full texts. Before each phase, the senior reviewer (CCMP) trained the reviewers with 10% of the retrieved studies. In all phases, consensus was reached through discussion. During the screening processes, disagreements were resolved through consensus by the pair of reviewers and if not achieved, by consulting the senior reviewer (CCMP).

### Data collection and data items

Independent paired reviewers (SQN/LPS, SQN/AGP, and JRC/ACSO) extracted data using a spreadsheet created on Excel software. The expert reviewer (SQN) trained the reviewers before extraction, using 5% of the included studies to guarantee uniformity and consistency during data extraction. During the extraction process, the pair of reviewers discussed and solved disagreements by consensus. When a consensus was not reached, the expert reviewer gave the final vote (SQN). All reviewers involved in the data collection had previous experience in data extraction and authored at least one SR.

The extracted data included the following information: authors, the geographical location of the authors, the journal’s name, impact factor from the Journal of Citation Reports, the research topic, the language of the publication, the included studies’ design, the number of primary studies, the number of RCTs and NRSIs, the total number of patients, the number of studies included in the meta-analysis, the risk of bias tool and the presence of a meta-analysis. The research topic was grouped according to Mejare et al. [8]: treatment and prevention of dental caries (fluorides, restorations and sealants), plaque control and oral hygiene education (toothbrushing, antimicrobials, mouthwashes and education programs), anesthesia (solutions, techniques), endodontics (rotatory and manual techniques), treatment of oral lesions (ankyloglossia and mucositis), behavior management/anxiety (techniques and technologies), and dental eruption.

One reviewer (RAB), experienced in assessing the certainty of the evidence through the GRADE approach, extracted the certainty of the evidence. For data extraction, we considered only reviews that presented the certainty of the evidence through a GRADE evidence profile table or summary of findings (SoF) table. The following variables were extracted for each outcome: the intervention and comparison, type of outcome, the effect estimate and confidence interval (95%CI), the direction of the effect (favoring the intervention or control), the final rating for each GRADE domain (no problem, serious, or very serious [for risk of bias, inconsistency, indirectness, imprecision, publication bias]), upgrading the certainty for dose-response, large effect and residual confounders (for NRSI). The final certainty was extracted per outcome (high, moderate, low, and very low) [11]. The same reviewer cross-checked data extraction one month later to minimize extraction errors.

The corresponding authors of the included studies were contacted once through email to clarify details when required.

### Methodological quality

Independent and trained paired reviewers (SQN/LPS, AGP/LPS, and JRC/ACSO) assessed the methodological quality of the included reviews using the AMSTAR 2 tool [12]. The expert reviewer trained the reviewers and discussed any disagreements until they reached a consensus (likewise regarding the description for data extraction). At least one of the paired reviewers had previous experience with AMSTAR 2 and authored at least one methodological survey (SQN, AGP, JRC).

AMSTAR 2 has 16 items and four criteria, each judged as “yes” (no critical weakness), “partial yes” (non-critical weakness), “no” (critical flaw), and “no meta-analysis conducted.” We followed the seven critical items according to AMSTAR 2 to rate the final quality (2, 4, 7, 9, 11, 13 and 15) [12]. The final quality of each SR was “high” when there was no or one “non-critical weakness” on one of the critical item; “moderate” when there was more than one “non-critical weakness;” “low” when there was one “critical” flaw with or without a “non-critical weaknesses,” or “critically low” when there was more than one “critical flaw” with or without “non-critical weaknesses.” For each AMSTAR 2 item, we grouped “yes” and “partial yes” into “no critical weaknesses” and “no” as “critical flaw.”

### Summary measurements and synthesis of results

The data were imported into IBM SPSS Statistics for Windows, Version 25 (Armonk, NY: IBM Corp.). Categorical variables were descriptively presented as absolute and relative frequencies, while numeric variables (e.g., total number of patients and included studies) were summarized using means and standard deviations. The Person chi-square test was used to test the association between methodological quality and the certainty of the evidence.

## Results

### Study selection

**Fig. 1:**
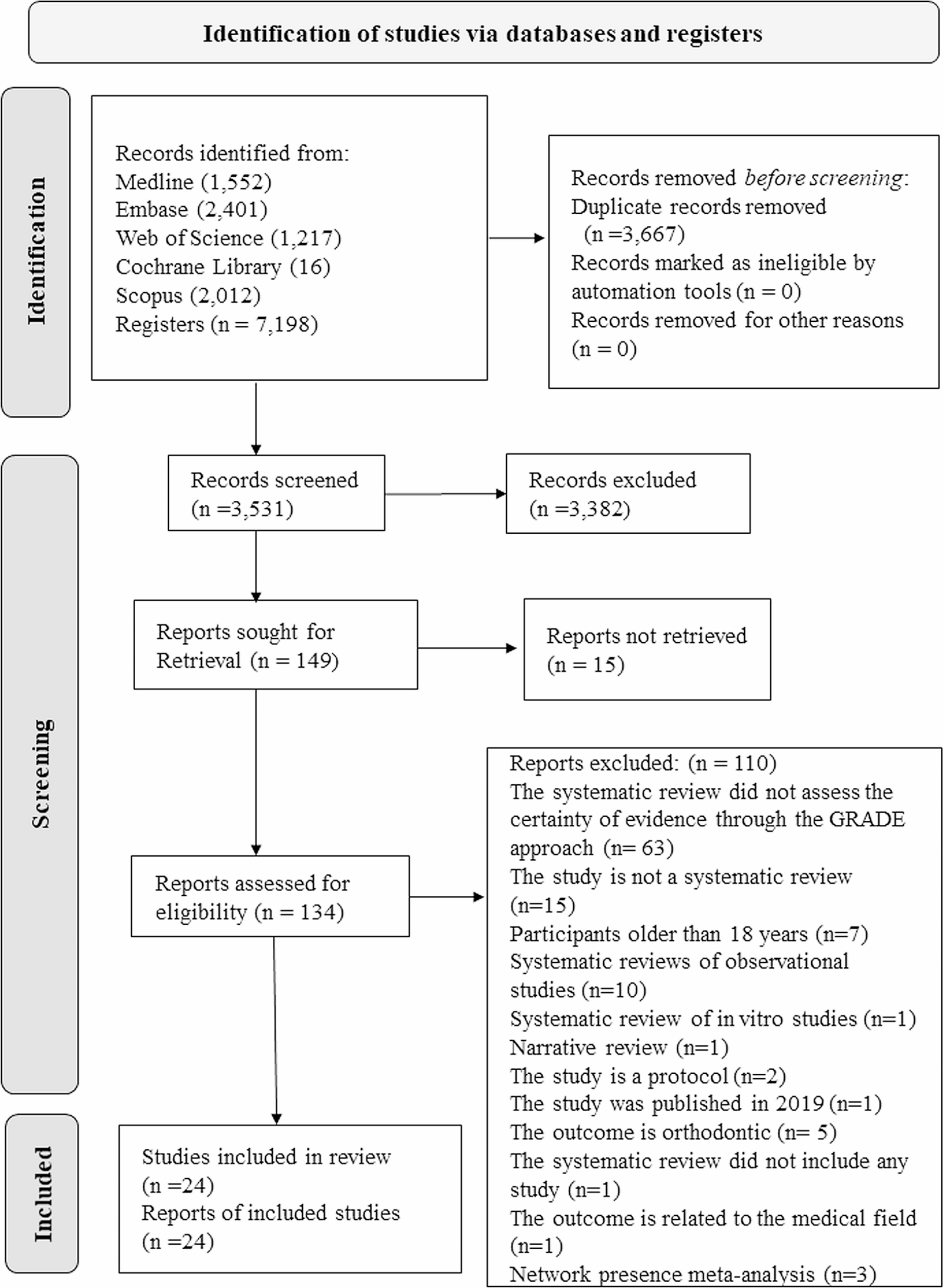
PRISMA flowchart showing the screening process

The electronic and manual searches retrieved 7,198 records. A total of 3,531 titles and abstracts of studies were selected after removing duplicates. One hundred thirty-four full texts were included for analysis. Finally, 24 studies that reported the certainty of the evidence through the GRADE approach were included (Fig. 1. PRISMA flowchart, Supplementary Table S2). Supplementary Table S3 shows the list of excluded studies and reasons for exclusion.

### Study characteristics

Table 1 shows the reviews’ characteristics. All reviews were published in English (100%), and one-third had authors from different countries collaborating with the research team (33.3%). Only four of 24 were Cochrane reviews.

**Table 1:** Frequency distribution of reviews’ characteristics

Variable	*N* = 24 (100%)
**Continent**	
Collaboration of authors from more than one country Asia Latin America Europe North America Middle East Oceania Africa	8 (33.3) 6 (25.0) 5 (20.8) 2 (8.3) 1 (4.1) 1 (4.1) 1 (4.1) 0 (0.0)
Title of the Journal (Impact factor)*	
Cochrane Database of Systematic Reviews (8.4) International Journal of Paediatric Dentistry (3.8) Pediatric Dentistry (1.6) Journal of Evidence-Based Dental Practice (3.6) Archives of Oral Biology (3.0) Clinical Oral Investigation (3.4) European Archives of Paediatric Dentistry (2.2) Evidence-Based Complementary and Alternative Medicine (2.650) International Endodontic Journal (5.0) Journal of Dentistry (4.4) Journal of Indian Society of Pedodontics and Preventive Dentistry (NR) Otolaryngology - Head Neck Surgery (3.4) Oral Diseases (3.8) Psychology & Health (3.3) Scientific Reports (4.6) Journal of the American Dental Association (3.9)	4 (16.6) 3 (12.5) 3 (12.5) 2 (8.3) 1 (4.1) 1 (4.1) 1 (4.1) 1 (4.1) 1 (4.1) 1 (4.1) 1 (4.1) 1 (4.1) 1 (4.1) 1 (4.1) 1 (4.1) 1 (4.1)
**Language of publication**	
English	24 (100.0)
**Number of included studies in the systematic review**	
Mean (SD) Minimum Maximum Total	12.4 (7.6) 4 35 322
**Number of RCTs included in the systematic reviews** (***n*** = **24 reviews that included RCTs)**	
Mean (SD) Minimum Maximum Total	10.5 (4.9) 4 26 252
**Number of NRSIs included in the systematic reviews** (***n*** = **4 reviews included NRSIs)**	
Mean (SD) Minimum Maximum Total	12.0 (11.3) 2 28 48
**Total number of patients**	
Mean (SD) Minimum Maximum Total	1,721.95 (1,670.56) 199 6,043 34,439
**Presence of meta-analysis?**	
Yes No	19 (79.2) 5 (20.8)
**Number of studies included in the meta-analysis**	
Mean (SD) Minimum Maximum Total	7.9 (4.0) 2 19 167
**Tool used to assess the risk of bias of RCTs**	
Conventional Cochrane risk of bias tool for RCTs Cochrane risk of bias tool for randomized studies (RoB 2)	14 (58.3) 10 (41.6)
**Tool used to assess the risk of bias of NRSIs**	
ROBINS-I MINORS RoB 2	2 (50.0) 1 (25.0) 1 (25.0)

RCTs: non-randomized controlled trials; NRSIs: non-randomized studies of interventions. NR: not reported; SD: standard deviation

*Impact factor extracted from Journal of Citation Reports on November 9^th^, 2023

The SRs included a mean of 12.4 original studies (standard deviation – SD: 7.6). All SRs included RCTs and four SRs included NRSIs and RCTs. The mean number of RCTs included was 10.5 (SD: 4.9). Nineteen reviews had meta-analysis (79.2%).

All the reviews used the original Cochrane Risk of Bias tool (58.3%) or the RoB 2 (41.6%) for the risk of bias of RCTs. Two reviews that included NRSIs used the ROBINS-I, one used MINORS and one used RoB 2.

### Methodological quality of included reviews

Table 2 shows the distribution of the methodological quality per research topic. The methodological quality assessed by AMSTAR 2 was high (*n* = 4, 16.7%), moderate (*n* = 3; 12.5%), low (*n* = 9, 37.5%), and critically low (*n* = 8, 33.3%). The most explored topic was the treatment and prevention of dental caries [11 reviews: 36.4% being critically low (*n* = 4), 54.5% low (*n* = 6), and one moderate quality], followed by plaque control and oral hygiene education (four reviews: one being critically low, one low, one moderate and one with high methodological quality).

**Table 2:** Distribution of methodological quality (AMSTAR 2) according to the research topic

Research Topic	Riks of bias (AMSTAR 2)				Total *N* (%)
	Critically low *N* (%)	Low *N* (%)	Moderate *N* (%)	High *N* (%)	
Treatment and prevention of dental caries	4 (36.4)	6 (54.5)	1 (9.1)	0 (0.0)	11 (100.0)
Plaque control and oral hygiene education	1 (25.0)	1 (25.0)	1 (25.0)	1 (25.0)	4 (100.0)
Anesthesia	1 (33.3)	1 (33.3)	0 (0.0)	1 (33.3)	3 (100.0)
Endodontics	0 (0.0)	1 (50.0)	0 (0.0)	1 (50.0)	2 (100.0)
Treatment of oral lesions	2 (100.0)	0 (0.0)	0 (0.0)	0 (0.0)	2 (100.0)
Behavior management /anxiety	0 (0.0)	0 (0.0)	1 (100.0)	0 (0.0)	1 (100.0)
Dental eruption	0 (0.0)	0 (0.0)	0 (0.0)	1 (100.0)	1 (100.0)
**Total**	8 (33.3)	9 (37.5)	3 (12.5)	4 (16.7)	24 (100.0)

RCTs: non-randomized controlled trials; NRSIs: non-randomized studies of interventions

Table 3 shows the quality assessed by AMSTAR 2. The majority of the reviews fulfilled most of the “no critical weaknesses” of AMSTAR 2. More than 90% of the reviews reported the appropriate inclusion of PICO components in the research question and inclusion criteria (item 1, 95.8%); explained the selection of study designs for inclusion in the review (item 3, 91.6%); reported a comprehensive literature search (item 4, 95.8%); reported duplicate and independent screening of studies (item 5, 91.6%); included an adequate description of studies’ characteristics (item 8, 100.0%); used a satisfactory tool for assessing the risk of bias (item 9, 100.0%); considered the risk of bias when interpreting the results (item 13, 91.6%); discussed the observed heterogeneity in the review results (item 14, 91.6%); and reported conflict or interests and funding of the review (item 16, 91.6%). Conversely, 79.1% of the reviews did not report the funding source for the studies included in the SR (item 10). The following items were responsible for the low or critically low quality of the reviews: item 2) 29.1% of reviews did not report the protocol before conducting the review nor deviations from the protocol; item 7) 45.8% of the reviews did not provide the list of excluded studies nor reasons for exclusion; item 15) 25.0% of reviews did not investigate publication bias nor explored its impact upon the results of the review.

**Table 3:** Frequency distribution of AMSTAR 2 items among reviews

AMSTAR 2 items	*N* = 24 (100%)
1. Did the research questions and inclusion criteria for the review include the components of PICO?	
No critical weaknesses Critical flaw	23 (95.8) 1 (4.1)
2-Did the report of the review contain an explicit statement that the review methods were established prior to conduct of the review and did the report justify any significant deviations from the protocol? *	
No critical weaknesses Critical flaw	17 (70.8) 7 (29.1)
3-Did the review authors explain their selection of the study designs for inclusion in the review?	
No critical weaknesses Critical flaw	22 (91.6) 2 (8.3)
4-Did the review authors use a comprehensive literature search strategy? *	
No critical weaknesses Critical flaw	23 (95.8) 1 (4.1)
5-Did the review authors perform study selection in duplicate?	
No critical weaknesses Critical flaw	22 (91.6) 2 (8.3)
6-Did the review perform data extraction in duplicate?	
No critical weaknesses Critical flaw	20 (83.3) 4 (16.6)
7-Did the review authors provide a list of excluded studies and justify the exclusions? *	
No critical weaknesses Critical flaw	13 (54.1) 11 (45.8)
8-Did the review authors describe the included studies in adequate detail?	
No critical weaknesses Critical flaw	24 (100.0) 0 (0.0)
9-Did the review authors use a satisfactory technique for assessing the risk of bias (RoB) in individual studies that were included in the review? *	
No critical weaknesses Critical flaw	24 (100.0)** 0 (0.0)
10- Did the review authors report on the sources of funding for the studies included in the review?	
No critical weaknesses Critical flaw	5 (20.8) 19 (79.1)
11-If meta-analysis was justified, did the review authors use appropriate methods for statistical combination of results? (Only complete this item if meta-analysis of other data synthesis techniques were reported)*	
No critical weaknesses Critical flaw No meta-analysis conducted	20 (83.3) 0 (0.0) 4 (16.6)
12- If meta-analysis was performed, did the review authors assess the potential impact of RoB in individual studies on the results of the meta-analysis or other evidence synthesis?	
No critical weaknesses Critical flaw No meta-analysis conducted	16 (66.6) 4 (16.6) 4 (16.6)
13- Did the review authors account for RoB in individual studies when interpreting/discussing the results of the review? *	
No critical weaknesses Critical flaw	22 (91.6) 2 (8.3)
14- Did the review authors provide a satisfactory explanation for, and discussion of, any heterogeneity observed in the results of the review?	
No critical weaknesses Critical flaw	22 (91.6) 2 (8.3)
15-If they performed quantitative synthesis, did the review authors carry out an adequate investigation of publication bias (small study bias) and discuss its likely impact on the results of the review? *	
No critical weaknesses Critical flaw No meta-analysis conducted	17 (70.8) 6 (25.0) 1 (4.1)
16-Did the review authors report any potential sources of conflict of interest, including any funding they received for conducting the review?	
No critical weaknesses Critical flaw	22 (91.6) 2 (8.3)

RoB: risk of bias

No critical weaknesses: yes and partial yes. Critical flaw: no answer

*Critical items according to AMSTAR 2

**One study used RoB 2 for RCTs and NRSIs. However, we considered partially fulfilling the criteria [13]

### Reporting the certainty of the evidence through the GRADE approach

Among 24 SRs that reported the GRADE approach, four SRs were excluded from the analysis, as two reviews did not present a GRADE evidence profile or SoF table with the certainty of the evidence [14, 15]; one review assessed the certainty per study (not per outcome) [16]; and one review combined RCTs and NRSIs to generate the evidence [17]. Consequently, we analyzed the certainty of the evidence for 20 reviews reporting 125 outcomes (112 outcomes generated from RCTs and 13 from NRSIs). Mainly, the certainty was low (33.1% and 7.7%) and very low (40.2% and 84.6%) for outcomes from RCTs and NRSIs, respectively (Tables 4 and 5).

**Table 4:** Distribution of the certainty of the evidence according to the research topic

Research Topic	Certainty of the evidence (GRADE approach)				Total *N* (%)
	High *N* (%)	Moderate *N* (%)	Low *N* (%)	Very Low *N* (%)	
**RCTs**					
Treatment and prevention of dental caries	0 (0.0)	3 (6.3)	23 (47.9)	22 (45.8)	48 (100.0)
Plaque control and oral hygiene education	0 (0.0)	3 (16.7)	7 (38.9)	8 (44.4)	18 (100.0)
Anesthesia	2 (13.3)	0 (0.0)	2 (13.3)	11 (73.3)	15 (100.0)
Endodontics	1 (7.7)	8 (61.5)	4 (30.8)	0 (0.0)	13 (100.0)
Treatment of oral lesions	0 (0.0)	2 (66.7)	1 (33.3)	0 (0.0)	3 (100.0)
Behavior management /anxiety	7 (63.6)	4 (36.4)	0 (0.0)	0 (0.0)	11 (100.0)
Dental eruption	0 (0.0)	0 (0.0)	0 (0.0)	4 (100.0)	4 (100.0)
**Total**	10 (8.9)	20 (17.9)	37 (33.0)	45 (40.2)	112 (100.0)
**NRSIs**					
Treatment and prevention of dental caries	0 (0.0)	1 (7.7)	1 (7.7)	11 (84.6)	13 (100.0)
**Total**	0 (0.0)	1 (7.7)	1 (7.7)	11 (84.6)	13 (100.0)

RCTs: non-randomized controlled trials; NRSIs: non-randomized studies of interventions

**Table 5:** Distribution of the certainty of the evidence (final rating of GRADE approach) according to the methodological quality judged by AMSTAR 2

Final rating of AMSTAR 2	Certainty of the evidence (GRADE approach)				Total *N* (%)
	High *N* (%)	Moderate *N* (%)	Low *N* (%)	Very Low *N* (%)	
**RCTs** ^†^					
High	0 (0.0)	**6 (24.0)** ^**‡‡**^	6 (24.0)	13 (52.0)	25 (100.0)
Moderate	**7 (43.8)** ^**‡**^	**4 (25.0)**	1 (6.3)	4 (25.0)	16 (100.0)
Low	3 (6.3)	9 (18.8)	18 (37.5)	18 (37.5)	48 (100.0)
Critically low	0 (0.0)	1 (4.3)	12 (52.2)	10 (43.5)	23 (100.0)
**Total**	10 (8.9)	20 (17.9)	37 (33.0)	45 (40.2)	112 (100.0)
**NRSIs** ^**††**^					
Low	0 (0.0)	1 (50.0)	1 (50.0)	0 (0.0)	2 (100.0)
Critically	0 (0.0)	0 (0.0)	0 (0.0)	11 (100.0)	11 (100.0)
**Total**	0 (0.0)	1 (7.7)	1 (7.7)	11 (84.6)	13 (100.0)

Pearson chi-square test: ^†^RCTs: *p* < 0.001; ^††^NRSIs: *p* < 0.002. Bold values show the high and moderate certainty for the high and moderate-quality reviews

^‡‡^Efficacy of virtual reality glasses versus control for behavior, anxiety, and pain management (3 outcomes) [18]. ^‡‡^Efficacy of rotatory instrument versus manual instrumentation for pain at 6 and 48 h of follow-up and instrumentation time (3 outcomes) [19]. Efficacy of theory-guided intervention versus *conven*tional education sessions for oral health behaviors (1 outcome) [20]. The 95%CI crossed the null effect line for the other high to moderate evidence

There were only 10 high-certainty outcomes (8.9% - RCT evidence) (Table 4). Among them, six favored the intervention: virtual reality eyeglasses were more effective than other distraction techniques for anxiety during restoration, behavior during caries removal, pain during restoration (behavior management/anxiety topic) [18], quality of obturation favoring the rotatory instrument versus manual (endodontics topic) [21], and less pain for articaine versus lidocaine (anesthesia topic) [22]. Another four high-certainty outcomes favored the intervention or were similar to the comparison group, in other words, the 95%CI crossed the null effect line though the effect estimated favored the intervention (behavior management/ anxiety topic) [18].

Yet, 20 outcomes were considered moderate (17.9%), and 10 showed more efficacy of the intervention than the comparison group: pain, instrumentation time [19, 21], and quality of obturation favoring rotatory instrument (endodontics topic) [21]; microorganisms count favoring synthetic antimicrobial [23]; dental plaque favoring modified toothbrushes (plaque control and oral hygiene education) [13]; and control of radio/chemotherapy-induced oral mucositis favoring honey (treatment of oral lesions) [24]. The remaining outcomes had CIs crossing the null effect line, indicating superior or similar efficacy favoring the intervention (Table 4).

Among NRSIs, there was only one moderate-certainty outcome (gingival health for rehabilitation with zirconia crown compared to stainless steel crown [treatment and prevention of dental caries]) [25]. Supplementary Table S4 lists the outcomes with very low, low, moderate and high certainty, the effect estimates with respective 95%CI, and the direction of the effect.

Table 5 shows the association between the methodological quality - as measured by AMSTAR 2 - and the certainty of the evidence – as measured by the GRADE approach. Based on Pearson chi-square tests, there was a statistically significant association between methodological quality and the certainty of the evidence for RCTs (*p* < 0.001) and NRSIs (*p* < 0.002).

Risk of bias was the main reason for downgrading the certainty of the evidence (serious problem: 55.4% among RCTs and 84.6% among NRSIs), followed by imprecision (53.5% among RCTs and 100% of NRSIs) (Table 6). There were serious problems of inconsistency (18.8% and 23.1% of the outcomes generated from RCTs and NRSIs) and serious problems of indirectness in 17.8% of outcomes from RCTs.

**Table 6:** Frequency and absolute distribution of overall and per domain GRADE rating evaluated per outcome (N: number of outcomes)

Certainty of evidence	RCTs	NRSTs
	*N* = 112 (100%)	*N* = 13 (100%)
High	10 (8.9)	0 (0.00)
Moderate	20 (17.8)	1 (7.7)
Low	37 (33.1)	1 (7.7)
Very low	45 (40.2)	11 (84.6)
**PER DOMAIN**		
**Risk of bias**		
No problem	34 (30.4)	2 (15.4)
Serious	62 (55.4)	11 (84.6)
Very serious	15 (13.4)	0 (0.0)
Not reported	01 (0.8)	0 (0.0)
**Inconsistency**		
No problem	77 (68.7)	10 (76.9)
Serious	21 (18.8)	3 (23.1)
Very serious	0 (0.0)	0 (0.0)
Not reported/Not applicable	14 (12.5)	0 (0.0)
**Indirectness**		
No problem	86 (76.8)	13(100.0)
Serious	20 (17.8)	0 (0.0)
Very serious	1 (0.9)	0 (0.0)
Not reported	5 (4.5)	0 (0.0)
**Imprecision**		
No problem	34 (30.3)	0 (0.0)
Serious	60 (53.5)	13 (100.0)
Very serious	16 (14.3)	0 (0.0)
Not reported	2 (1.9)	0 (0.0)
**Publication bias**		
Not suspected	96 (85.8)	13(100.0)
Suspected Not reported	8 (7.1) 8 (7.1)	0 (0.0) 0 (0.0)
**Dose response**		
Not reported	NA	0 (0.0)
Not upgrade	NA	13 (100.0)
Upgrade	NA	0 (0.0)
Not apply	112 (100.0)	0 (0.0)
**Magnitude of the effect**		
Not reported	NA	0 (0.0)
Not upgrade	NA	13 (100.0)
Upgrade	NA	0 (0.0)
Not apply	112 (100.0)	0 (0.0)
**Residual confounders**		
Not reported	NA	0 (0.0)
Not upgrade	NA	13 (100.0)
Upgrade	NA	0 (0.0)
Not apply	112 (100.0)	0 (0.0)

RCTs: non-randomized controlled trials; NRSIs: non-randomized studies of interventions. NA: not apply

The certainty of the evidence was not upgraded for any outcome (NRSIs evidence).

## Discussion

This survey found the methodological quality of systematic reviews in pediatric dentistry was mostly low or very low. Another survey evaluating pediatric dentistry reviews found similar results. That study, published in 2015, found a 53% high risk of bias in SRs [8]. However, the low methodological quality is not an exclusive characteristic of pediatric dentistry SRs. Other dentistry, orthodontics, and periodontology surveys also considered the SRs in these areas mainly low and critically low quality, using AMSTAR 2 [4–6]. Similar findings are also common in medicine, such as post-interventions after breast cancer, treatment of major depression, and living SRs of COVID-19 – all presenting mainly low and critically low quality [26–28]. Even the updated SR does not seem to improve compared to the original one, as found by a study comparing the quality of the original and updated non-Cochrane reviews. That survey found that all original SRs were low and critically low quality, and there was no significant difference in the methodological quality comparing the original SRs with the updated version [29]. Other surveys found that Cochrane reviews had higher quality than non-Cochrane reviews [1, 2]. However, these surveys date from seven years ago. Another survey included SRs in dentistry, published from 1980 to 2014, and concluded that the methodological quality increased over the years, especially after the 2000’s [30].

Four AMSTAR 2 items were the most common sources of methodological problems (items 2, 7, 10 and 15). Three of these are considered “critical” items that might have influenced the low and critically low quality (2, 7 and 15). Around one-third of reviews did not report the protocol before conducting the review (item 2), indicating a general neglect of the research protocol. Documenting the protocol before conducting the review is important to minimize potential bias during the review process. For example, prior knowledge of the evidence may influence the definition of a systematic question, the choice for eligibility criteria, or the prespecification of intervention comparisons and outcome analysis [31]. We speculate that deviation from the protocol may be higher than reported. Another critical concern is simple to cover but neglected, as indicated in item 7: 45.8% of reviews did not provide the list of excluded studies with reasons. Authors could publish lists of excluded studies in the accompanying supplementary material. Lastly, about one-quarter of reviews failed to analyze the publication bias and its impact on the results (item 15). Moreover, item 10 could help address item 15. Item 10 relates to reporting the funding sources of the included studies in the SR. The funding source of the included studies in the SR can help resolve conflicts of interest and publication bias. 79% of SRs did not report the funding sources of included studies within the SR. A previous study found that many meta-analyses in pediatric dentistry did not adequately assess the possibility and impact of reporting biases, especially publication bias, which might influence results and conclusions [32]. Funnel plots, Begg’s and Egger’s tests, and identifying for-profit organizations associated with studies can help to reveal publication bias. The GRADE approach suggests downgrading the certainty of the evidence in these situations [33]. The investigation of industry funding in medicine meta-analysis results is better recognized [34, 35] but still lacking in dentistry [32, 36]. Coincidence or not, we found that only 7.1% of outcomes that originated from RCTs had suspected publication bias (or someone could speculate that the reviews failed to analyze and identify publication bias properly). In addition, it is important to highlight that the funding of included studies (item 10) differs from funding and conflict of interest of the systematic review (item 16). The review adequately addressed this last item (item 16 was covered by 91.6% of reviews). To summarize, for-profit funding should be investigated in SRs of interventions.

The certainty of the evidence was generally low and very low, and the main problems for downgrading the certainty of the evidence were due to risk of bias and imprecision. About 19% of SRs in dentistry published in 2017 reported the GRADE approach [37]. In orthodontics and periodontology, the proportion of SRs of interventions that included the GRADE approach was 54.3% and 25.2%, respectively [4, 5]. A 2015 systematic map of pediatric dentistry reviews reported that many studies did not use the GRADE approach, in agreement with our study [8]. Nine years separated our survey from the previous one, and still, the proportion of pediatric dentistry reviews using the GRADE approach is low. We excluded 63 SRs because they did not include the GRADE approach. Therefore, we could estimate around 28% of SRs reporting the GRADE approach in the present study [(24 of (63 + 24 = 87), Fig. 1]. On a positive note, of those SRs that incorporated the GRADE approach, 83.3% followed the GRADE criteria through a GRADE evidence profile or SoF Table (20 of 24).

Similar to another survey in dentistry, the main problems for downgrading the certainty were risk of bias and imprecision [2]. The SRs included the proper risk of bias tools according to the study design, as the RoB 2 and ROBINS-I were predominantly used for RCTs and NRSIs, respectively. Also, when correctly assessed, the risk of bias may facilitate the assessment of the certainty of the evidence. Similarly, imprecision might be easily detected, especially for evidence generated from meta-analysis where the 95%CI crosses the null effect line. Notably, about 50% of the moderate evidence was downgraded due to imprecision because the 95%CI crossed the null effect line. In this case, it means moderate evidence that the intervention was similar to or superior to the control. However, caution should be taken, as the authors did not evaluate the evidence based on the magnitude of the effect or based on the minimal important difference, as recently recommended by the GRADE approach [38].

Ten outcomes had high certainty of evidence. It means that the true effect lies very close to the observed effect, and future research can hardly change the evidence [11]. However, 4 of 10 high-certainty outcomes had the 95%CIs crossing the null effect line. This imprecision means that the intervention and the control have similar effects. Reliable evidence for decision-making should consider the following criteria: (1) the magnitude of the effect or if the desirable health effect (or benefit) is substantial; (2) if the undesirable health effect (or harms) is substantial; (3) the variability or uncertainty about the values (confidence interval addressing imprecision); (4) the certainty of the evidence [39]; and we would add (5) whether the evidence is generated from high-quality SR - or at least moderate-quality SR. We found 17 high-to-moderate certainty of evidence outcomes from high-to-moderate-quality reviews (15%, Table 5). The most reliable evidence for decision-making was the moderate-quality SR that reported high certainty of the evidence for virtual reality glasses for behavior, anxiety, and pain management (3 outcomes) [18]. Similarly, there is moderate certainty for the efficacy of rotatory instruments versus manual instruments at 6 and 48h of follow-up and instrumentation time (3 outcomes) [19] and moderate certainty of the efficacy of the theory-guided intervention compared to conventional education session for oral hygiene education (1 outcome) [20] – these outcomes were from high-quality reviews. The other high and moderate certainty outcomes had 95%CI crossing the null effect line or were from SRs presenting low or critically low methodological quality. Likewise, all NRSIs presented low or critically low quality, though one outcome contained moderate certainty of evidence. Our results are similar to a previous methodological survey that found mainly low or very low evidence in pediatric dentistry SRs. In that survey, 15 SRs reported high to moderate certainty of evidence for fluoride technologies (or other technologies) for caries prevention [8]. Contrary to the previous study, we did not find high or moderate evidence for the treatment and prevention of dental caries.

In summary, low and very low evidence were prominent in this survey. Regardless of whether the outcome had problems of risk of bias, inconsistency, indirectness, imprecision or publication bias, it is highly probable that future research will change the evidence published by these SRs [11]. Therefore, future clinical trials and reviews in these areas are welcome.

### Strengths and limitations

The study is strong in reporting the certainty per outcome and per GRADE domain. Furthermore, we analyzed the methodological quality and the certainty of the evidence per research topics, bringing to light the new areas in pediatric dentistry where new research is welcome and highlighting the high and moderate certainty outcomes in the area. In our view, all the reviews included here follow the criteria to be considered a SR: clear clinical question, clear systematic search, reproducible methodology, risk of bias, and systematic presentation of results [6, 10].

There are limitations, such as excluding reviews without the certainty of the evidence to compare quality. Therefore, the reviews analyzed here may not necessarily be fully representative of all pediatric dentistry-related interventions, as we used a convenience sample. The convenience sample of reviews published at a two-year interval limits the external validity. Likewise, our results do not fully represent the Cochrane reviews due to their low number in our sample. Due to the possible overlap of pediatric dentistry and other specialties, there is the possibility of exclusion of SRs covering pediatric dentistry and other areas.

The certainty of the evidence represents the judgment of the review authors, as we did not cross-check their decision. Finally, we did not judge the minimal important difference and magnitude of the effect of the evidence generated.

### Perspectives for future research and clinical practice

Future systematic reviews of interventions should require the certainty of the evidence. Furthermore, the authors would benefit from the magnitude of the effect, minimal important difference, and GRADE guideline 26 to interpret the evidence [38, 40]. Our results can help clinicians find high and moderated-quality evidence in pediatric dentistry and help researchers evaluate which low and very low-quality evidence is worthy of research in the future.

## Conclusion

Most reviews fulfilled the majority of AMSTAR 2 criteria despite the mainly low and critically low final quality. The proportion of studies assessing the certainty of the evidence through the GRADE approach was small. The main problems of the certainty of the evidence were due to risk of bias and imprecision. Considering that the greatest evidence of these reviews was mainly low and very low, there is space for future clinical trials and SRs in pediatric dentistry.

### Registration and protocol

The protocol was registered *a priori* in the International Prospective Register of Systematic Reviews (PROSPERO #CRD42022365443). There have been no modifications regarding the protocol methods since the start of this overview.

### Electronic supplementary material

Below is the link to the electronic supplementary material.

Supplementary Material 1

Supplementary Material 2

Supplementary Material 3

Supplementary Material 4

## Data Availability

All data generated or analyzed during this study are included in this published article as Supplementary Table S4.

## Abbreviations

AMSTAR 2: Assessing the Methodological Quality of Systematic Reviews
CI: Confidence interval
GRADE: Grading of Recommendations, Assessment, Development and Evaluation
NRSI: Non-randomized study of intervention
PRISMA: Preferred Reporting Items for Systematic Reviews and Meta-Analyses
RCT: Randomized controlled trial
SR: Systematic review

## References

[1] Smail-Faugeron V, Fron-Chabouis H, Courson F (2014). Methodological quality and implications for practice of systematic Cochrane reviews in pediatric oral health: a critical assessment. BMC Oral Health.

[2] Pandis N, Fleming PS, Worthington H, Salanti G (2015). The quality of the evidence according to GRADE is predominantly low or very low in oral health systematic reviews. PLoS ONE.

[3] Fleming PS, Koletsi D, Ioannidis JP, Pandis N (2016). High quality of the evidence for medical and other health-related interventions was uncommon in Cochrane systematic reviews. J Clin Epidemiol.

[4] Notaro SQ, Hermont AP, Cruz PV, Maia RM, Avila WM, Poklepovic Pericic T, Abreu LG, Jiao R, Martins-Pfeifer C (2024). Methodological quality of systematic reviews addressing orthodontic interventions: methodological study. Pesq Bras Odontop Clin Int.

[5] Pereira AG, Martins CC, Campos JR, Faria SF, Notaro SQ, Poklepovic-Pericic T, Costa LC, Costa FO, Cota LO (2023). Critical appraisal of systematic reviews of intervention studies in periodontology using AMSTAR 2 and ROBIS tools. J Clin Exp Dent.

[6] Pauletto P, Polmann H, Reus JC, de Oliveira JMD, Chaves D, Lehmkuhl K, Massignan C, Stefani CM, Martins CC, Flores-Mir C, De Canto L. G: Critical appraisal of systematic reviews of intervention in dentistry published between 2019–2020 using the AMSTAR 2 tool. Evid Based Dent 2022. 10.1038/s41432-022-0802-5 36104402

[7] Atkins D, Best D, Briss PA, Eccles M, Falck-Ytter Y, Flottorp S, Guyatt GH, Harbour RT, Haugh MC, Henry D (2004). Grading quality of evidence and strength of recommendations. BMJ.

[8] Mejare IA, Klingberg G, Mowafi FK, Stecksen-Blicks C, Twetman SH, Tranaeus SH (2015). A systematic map of systematic reviews in pediatric dentistry–what do we really know?. PLoS ONE.

[9] Page MJ, Moher D, Bossuyt PM, Boutron I, Hoffmann TC, Mulrow CD, Shamseer L, Tetzlaff JM, Akl EA, Brennan SE (2021). PRISMA 2020 explanation and elaboration: updated guidance and exemplars for reporting systematic reviews. BMJ.

[10] Higgins JPT, Thomas J, Chandler J, Cumpston M, Li T, Page MJ. Welch VAe: Cochrane Handbook for Systematic Reviews of Interventions version 6.4 (updated August 2023). Cochrane, 2023. www.training.cochrane.org/handbook.

[11] Guyatt GH, Oxman AD, Vist GE, Kunz R, Falck-Ytter Y, Alonso-Coello P, Schunemann HJ (2008). Group GW: GRADE: an emerging consensus on rating quality of evidence and strength of recommendations. BMJ.

[12] Shea BJ, Reeves BC, Wells G, Thuku M, Hamel C, Moran J, Moher D, Tugwell P, Welch V, Kristjansson E, Henry DA (2017). AMSTAR 2: a critical appraisal tool for systematic reviews that include randomised or non-randomised studies of healthcare interventions, or both. BMJ.

[13] Lai YYL, Zafar S, Leonard HM, Walsh LJ, Downs JA (2022). Oral health education and promotion in special needs children: systematic review and meta-analysis. Oral Dis.

[14] Davidovich E, Shafir S, Shay B, Zini A (2020). Plaque removal by a Powered Toothbrush Versus a Manual Toothbrush in children: a systematic review and Meta-analysis. Pediatr Dent.

[15] Santos GM, Pacheco RL, Bussadori SK, Santos EM, Riera R, de Oliveira Cruz Latorraca C, Mota P, Benavent Caldas Bellotto EF, Martimbianco ALC (2020). Effectiveness and safety of ozone therapy in Dental Caries Treatment: systematic review and Meta-analysis. J Evid-Based Dent Pract.

[16] Khan U, MacPherson J, Bezuhly M, Hong P (2020). Comparison of Frenotomy techniques for the treatment of Ankyloglossia in children: a systematic review. Otolaryngol Head Neck Surg.

[17] Kamber R, Meyer-Lueckel H, Kloukos D, Tennert C, Wierichs RJ (2021). Efficacy of sealants and bonding materials during fixed orthodontic treatment to prevent enamel demineralization: a systematic review and meta-analysis. Sci Rep.

[18] Custódio NB, Costa FS, Cademartori MG, Costa VPP, Goettems ML (2020). Effectiveness of virtual reality glasses as a distraction for children during Dental Care. Pediatr Dent.

[19] Manchanda S, Sardana D, Yiu CKY (2020). A systematic review and meta-analysis of randomized clinical trials comparing rotary canal instrumentation techniques with manual instrumentation techniques in primary teeth. Int Endod J.

[20] Xiang B, Wong HM, Perfecto AP, McGrath CPJ (2021). The application of theory-guided oral health interventions in adolescents: a systematic review and meta-analysis of randomized controlled trials. Psychol Health.

[21] Chugh VK, Patnana AK, Chugh A, Kumar P, Wadhwa P, Singh S (2021). Clinical differences of hand and rotary instrumentations during biomechanical preparation in primary teeth-A systematic review and meta-analysis. Int J Paediatr Dent.

[22] Taneja S, Singh A, Jain A (2020). Anesthetic effectiveness of Articaine and Lidocaine in Pediatric patients during Dental procedures: a systematic review and Meta-analysis. Pediatr Dent.

[23] Martins ML, Ribeiro-Lages MB, Masterson D, Magno MB, Cavalcanti YW, Maia LC, Fonseca-Goncalves A (2020). Efficacy of natural antimicrobials derived from phenolic compounds in the control of biofilm in children and adolescents compared to synthetic antimicrobials: a systematic review and meta-analysis. Arch Oral Biol.

[24] Hao S, Ji L, Wang Y. Effect of Honey on Pediatric Radio/Chemotherapy-Induced oral mucositis (R/CIOM): a systematic review and Meta-analysis. Evid-Based Complement Alternat Med; 2022. ID 6906439. 10.1155/2022/6906439 PMC8956378 35341151

[25] Patnana AK, Chugh VK, Chugh A, Vanga NRV, Kumar P (2022). Effectiveness of zirconia crowns compared with stainless steel crowns in primary posterior teeth rehabilitation: a systematic review and meta-analysis. J Am Dent Assoc.

[26] Olsson Moller U, Beck I, Ryden L, Malmstrom M (2019). A comprehensive approach to rehabilitation interventions following breast cancer treatment - a systematic review of systematic reviews. BMC Cancer.

[27] Matthias K, Rissling O, Pieper D, Morche J, Nocon M, Jacobs A, Wegewitz U, Schirm J, Lorenz RC (2020). The methodological quality of systematic reviews on the treatment of adult major depression needs improvement according to AMSTAR 2: a cross-sectional study. Heliyon.

[28] Luo J, Chen Z, Liu D, Li H, He S, Zeng L, Yang M, Liu Z, Xiao X, Zhang L (2023). Methodological quality and reporting quality of COVID-19 living systematic review: a cross-sectional study. BMC Med Res Methodol.

[29] Gao Y, Cai Y, Yang K, Liu M, Shi S, Chen J, Sun Y, Song F, Zhang J, Tian J (2020). Methodological and reporting quality in non-cochrane systematic review updates could be improved: a comparative study. J Clin Epidemiol.

[30] Wasiak J, Shen AY, Tan HB, Mahar R, Kan G, Khoo WR, Faggion CM (2016). Methodological quality assessment of paper-based systematic reviews published in oral health. Clin Oral Investig.

[31] Lasserson TJ, Thomas J, Higgins JPT, Toby J, Lasserson J, Thomas. Julian PT Higgins. In: Higgins JPT, Thomas J, Chandler J, Cumpston M, Li T, Page MJ, Welch VA, editors. Cochrane Handbook for Systematic Reviews of Interventions version 6.4 (updated August 2023). Cochrane, 2023. Chapter 1. www.training.cochrane.org/handbook.

[32] Papageorgiou SN, Dimitraki D, Coolidge T, Kotsanos N (2015). Publication bias & small-study effects in pediatric dentistry meta-analyses. J Evid-Based Dent Pract.

[33] Zhang Y, Akl EA, Schunemann HJ (2018). Using systematic reviews in guideline development: the GRADE approach. Res Synth Methods.

[34] Ahn RWA, Abraham A, Saba S, Korenstein D, Madden E, Boscardin WJ, Keyhani S (2017). Financial ties of principal investigators and randomized controlled trial outcomes: cross sectional study. BMJ.

[35] Amiri AR, Kanesalingam K, Cro S, Casey AT. Does source of funding and conflict of interest influence the outcome and quality of spinal research? J Spine *2*014, 14(2):308–14. 10.1016/j.spinee.2013.10.047 24231776

[36] Martins CC, Riva JJ, Firmino RT, Colunga-Lozano LE, Granville-Garcia AF, Zhang Y, Schünemann HJ (2019). Conflict of interest is not associated with positive conclusions in toothpaste trials: a systematic survey. J Clin Epidemiol.

[37] Bassani R, Pereira GKR, Page MJ, Tricco AC, Moher D, Sarkis-Onofre R (2019). Systematic reviews in dentistry: current status, epidemiological and reporting characteristics. J Dent.

[38] Carrasco-Labra A, Devji T, Qasim A, Phillips MR, Wang Y, Johnston BC, Devasenapathy N, Zeraatkar D, Bhatt M, Jin X (2021). Minimal important difference estimates for patient-reported outcomes: a systematic survey. J Clin Epidemiol.

[39] Schunemann HJ, Neumann I, Hultcrantz M, Brignardello-Petersen R, Zeng L, Murad MH, Izcovich A, Morgano GP, Baldeh T, Santesso N (2022). GRADE guidance 35: update on rating imprecision for assessing contextualized certainty of evidence and making decisions. J Clin Epidemiol.

[40] Santesso N, Glenton C, Dahm P, Garner P, Akl E, Alper B, Brignardello-Petersen R, Carrasco-Labra A, De Beer H, Hultcrantz M (2019). GRADE guidelines 26: informative statements to communicate the findings of systematic reviews of interventions. J Clin Epidemiol.

