# Supplementary material for: Pediatric dentistry systematic reviews using the GRADE approach: methodological study
Source: BMC Oral Health. 2024 Jul 13;24:787. doi: 10.1186/s12903-024-04542-w (PMC11245772; doi:10.1186/s12903-024-04542-w)
Supplement: Supplementary file 3 — Supplementary Material 3 [file 12903_2024_4542_MOESM3_ESM.docx]

**Title**

Pediatric dentistry systematic reviews using the GRADE approach: methodological study

**Authors**

Rachel Alvarenga-Brant, Sarah Queiroz Notaro, Cristine Miron Stefani, Graziela De Luca Canto, Alexandre Godinho Pereira, Luciana Póvoa-Santos, Ana Clara Souza-Oliveira, Julya Ribeiro Campos, Carolina Castro Martins-Pfeifer

**Supplementary material**

**Table S1.** Search strategies for each database. From January 1^st^ 2020 to March 3^rd^ 2022.

| **Medline through Ovid** [http://ovidsp.dc2ovid.ez27.periodicos.capes.gov.br/](about:blank) |
| --- |
| #1. pediatric.mp. or exp Pediatrics/ |
| #2. child*.mp. or exp Child/ |
| #3. infant*.mp. or exp Infant/ |
| #4. newborn.mp. or exp Infant, Newborn/ |
| #5. exp Adolescent/ or adolescents*.mp. |
| #6. teen*.mp. |
| #7. pediatric dentistry.mp. or exp Pediatric Dentistry/ |
| #8. oral health.mp. or exp Oral Health/ |
| #9. primary teeth.mp. or exp Tooth, Deciduous/ |
| #10. primary tooth.mp. or exp Tooth, Deciduous/ |
| #11. deciduous teeth.mp. or exp Tooth, Deciduous/ |
| #12. deciduous tooth.mp. or exp Tooth, Deciduous/ |
| #13. dental caries.mp. or exp Dental Caries/ |
| #14. Analysis of Variance.mp. or exp "Analysis of Variance"/ |
| #15. cavitated teeth.mp. |
| #16. cavitated tooth.mp. |
| #17. dental cavit*.mp. |
| #18. exp Tooth Injuries/ or dental trauma.mp. |
| #19. tooth trauma.mp. |
| #20. tooth fracture.mp. or exp Tooth Fractures/ |
| #21. teeth trauma.mp. |
| #22. tooth avulsion.mp. or exp Tooth Avulsion/ |
| #23. teeth avulsion.mp. |
| #24. dental restoration.mp. |
| #25. dental filling.mp. |
| #26. exp Composite Resins/ or composite resin*.mp. |
| #27. exp Compomers/ or compomer*.mp. |
| #28. exp Glass Ionomer Cements/ or glass ionomer cermet*.mp. |
| #29. dental crown*.mp. |
| #30. exp Tooth Replantation/ or tooth replantation.mp. |
| #31. teeth replantation*.mp. |
| #32. dental pulp.mp. or exp Dental Pulp/ |
| #33. exp Pulpectomy/ or exp "Pulp Capping and Pulpectomy Agents"/ or pulpectomy.mp. |
| #34. pulpotomy.mp. or exp Pulpotomy/ |
| #35. exp "Root Canal Therapy"/ or endodontic treatment*.mp. |
| #36. periapical diseases.mp. or exp Periapical Diseases/ |
| #37. surger*.mp. or exp Surgery, Oral/ |
| #38. systematic review*.mp. |
| #39.meta-analysis.mp. or exp Meta-Analysis/ |
| #40.network meta-analysis.mp. or exp Network Meta-Analysis/ or exp Meta-Analysis as Topic/ |
| #41. 1 or 2 or 3 or 4 or 5 or 6 |
| #42. 7 or 8 or 9 or 10 or 11 or 12 or 13 or 14 or 15 or 16 or 17 or 18 or 19 or 20 or 21 or 22 or 23 or 24 or 25 or 26 or 27 or 28 or 29 or 30 or 31 or 32 or 33 or 34 or 35 or 36 or 37 |
| #43. 38 or 39 or 40 |
| #44. 41 and 42 and 43 |
| #45. limit 44 to yr="2020 - 2022" |
| **Embase trought Ovid** [https://www-embase.ez27.periodicos.capes.gov.br/](about:blank) |
| (pediatric:ab,ti OR child:ab,ti OR infant*:ab,ti OR newborn:ab,ti OR adolescents*:ab,ti OR teen*:ab,ti) AND ('pediatric dentistry' OR 'oral health' OR 'primary teeth' OR 'primary tooth' OR 'deciduous teeth' OR 'deciduous tooth' OR 'dental caries' OR 'analysis of variance' OR 'cavitated teeth' OR 'cavitated tooth' OR 'dental cavit*' OR 'dental trauma' OR 'tooth trauma' OR 'tooth fracture' OR 'teeth trauma' OR 'tooth avulsion' OR 'teeth avulsion' OR 'dental restoration' OR 'dental filling' OR 'composite resin*' OR compomer* OR 'glass ionomer cement*' OR 'dental crown*' OR 'tooth replantation' OR 'teeth replantation*' OR 'dental pulp' OR pulpectomy OR pulpotomy OR 'endodontic treatment*' OR 'periapical diseases' OR surger*) AND ('systematic review*':ab,ti OR 'meta-analysis':ab,ti OR 'network meta-analysis':ab,ti) AND (2020:py OR 2021:py OR 2022:py) |
| **Web of Science** [http://apps-webofknowledge.ez27.periodicos.capes.gov.br/](about:blank) |
| TS=((Pediatric OR child OR infant* OR newborn OR adolescents* OR teen*) AND (“pediatric dentistry” OR “oral health” OR “primary teeth” OR “primary tooth” OR “deciduous teeth” OR “deciduous tooth” OR “dental caries” OR “analysis of variance” OR “cavitated teeth” OR “cavitated tooth” OR “dental cavit*” OR “dental trauma” OR “tooth trauma” OR “tooth fracture” OR “teeth trauma” OR “tooth avulsion” OR “teeth avulsion” OR “dental restoration” OR “dental filling” OR “composite resin*” OR compomer* OR “glass ionomer cement*” OR “dental crown*” OR “tooth replantation” OR “teeth replantation*” OR “dental pulp” OR pulpectomy OR pulpotomy OR “endodontic treatment*” OR “periapical diseases” OR surger*) AND (“systematic review*” OR “meta-analysis” OR “network meta-analysis”)) and 2022 or 2021 or 2020 (Years of publication) |
| **Scopus** [https://www-scopus.ez27.periodicos.capes.gov.br/](about:blank) |
| TITLE-ABS-KEY(pediatric OR child OR infant* OR newborn OR adolescents* OR teen*) AND  TITLE-ABS-KEY ( "pediatric dentistry"  OR  "oral health"  OR  "primary teeth"  OR  "primary tooth"  OR  "deciduous teeth"  OR  "deciduous tooth"  OR  "dental caries"  OR  "analysis of variance"  OR  "cavitated teeth"  OR  "cavitated tooth"  OR  "dental cavit*"  OR  "dental trauma"  OR  "tooth trauma"  OR  "tooth fracture"  OR  "teeth trauma"  OR  "tooth avulsion"  OR  "teeth avulsion"  OR  "dental restoration"  OR  "dental filling"  OR  "composite resin*"  OR  compomer*  OR  "glass ionomer cement*"  OR  "dental crown*"  OR  "tooth replantation"  OR  "teeth replantation*"  OR  "dental pulp"  OR  pulpectomy  OR  pulpotomy  OR  "endodontic treatment*"  OR  "periapical diseases"  OR  surger* )  AND  TITLE-ABS-KEY ( "systematic review*"  OR  "meta-analysis"  OR  "network meta-analysis" )  AND  ( LIMIT-TO ( PUBYEAR ,  2022 )  OR  LIMIT-TO ( PUBYEAR ,  2021 )  OR  LIMIT-TO ( PUBYEAR ,  2020 ) ) |
| **Cochrane Library** [https://www.cochranelibrary.com/](about:blank) |
| (Pediatric OR child OR infant* OR newborn OR adolescents* OR teen*) AND (“pediatric dentistry” OR “oral health” OR “primary teeth” OR “primary tooth” OR “deciduous teeth” OR “deciduous tooth” OR “dental caries” OR “analysis of variance” OR “cavitated teeth” OR “cavitated tooth” OR “dental cavit*” OR “dental trauma” OR “tooth trauma” OR “tooth fracture” OR “teeth trauma” OR “tooth avulsion” OR “teeth avulsion” OR “dental restoration” OR “dental filling” OR “composite resin*” OR compomer* OR “glass ionomer cement*” OR “dental crown*” OR “tooth replantation” OR “teeth replantation*” OR “dental pulp” OR pulpectomy OR pulpotomy OR “endodontic treatment*” OR “periapical diseases” OR surger*) AND (“systematic review*” OR “meta-analysis” OR “network meta-analysis”) in Title Abstract Keyword - with Cochrane Library publication date Between Jan 2020 and Feb 2022 (Word variations have been searched) |

**Table S2.** List of included studies.

| 1. | Manchanda S, Sardana D, Yiu CKY. A systematic review and meta-analysis of randomized clinical trials comparing rotary canal instrumentation techniques with manual instrumentation techniques in primary teeth. Int Endod J. 2020 Mar;53(3):333-353. doi: 10.1111/iej.13233. Epub 2019 Nov 26. PMID: 31587323. |
| --- | --- |
| 2. | Lam PPY, Sardana D, Ekambaram M, Lee GHM, Yiu CKY. Effectiveness of Pit and Fissure Sealants for Preventing and Arresting Occlusal Caries in Primary Molars: A Systematic Review and Meta-Analysis. J Evid Based Dent Pract. 2020 Jun;20(2):101404. doi: 10.1016/j.jebdp.2020.101404. Epub 2020 Jan 29. PMID: 32473795. |
| 3. | Martins ML, Ribeiro-Lages MB, Masterson D, Magno MB, Cavalcanti YW, Maia LC, Fonseca-Gonçalves A. Efficacy of natural antimicrobials derived from phenolic compounds in the control of biofilm in children and adolescents compared to synthetic antimicrobials: A systematic review and meta-analysis. Arch Oral Biol. 2020 Oct;118:104844. doi: 10.1016/j.archoralbio.2020.104844. Epub 2020 Jul 21. PMID: 32736143. |
| 4. | Kamber R, Meyer-Lueckel H, Kloukos D, Tennert C, Wierichs RJ. Efficacy of sealants and bonding materials during fixed orthodontic treatment to prevent enamel demineralization: a systematic review and meta-analysis. Sci Rep. 2021 Aug 16;11(1):16556. doi: 10.1038/s41598-021-95888-6. |
| 5. | Taneja S, Singh A, Jain A. Anesthetic Effectiveness of Articaine and Lidocaine in Pediatric Patients During Dental Procedures: A Systematic Review and Meta-Analysis. Pediatr Dent. 2020 Jul 15;42(4):273-281 |
| 6. | Chugh VK, Patnana AK, Chugh A, Kumar P, Wadhwa P, Singh S. Clinical differences of hand and rotary instrumentations during biomechanical preparation in primary teeth-A systematic review and meta-analysis. Int J Paediatr Dent. 2021 Jan;31(1):131-142. doi: 10.1111/ipd.12720. Epub 2020 Sep 23. |
| 7. | Khan U, MacPherson J, Bezuhly M, Hong P. Comparison of Frenotomy Techniques for the Treatment of Ankyloglossia in Children: A Systematic Review. Otolaryngol Head Neck Surg. 2020 Sep;163(3):428-443. doi: 10.1177/0194599820917619 |
| 8. | Smolarek PC, Wambier LM, Siqueira Silva L, Chibinski ACR. Does computerized anaesthesia reduce pain during local anaesthesia in paediatric patients for dental treatment? A systematic review and meta-analysis. Int J Paediatr Dent. 2020 Mar;30(2):118-135. doi: 10.1111/ipd.12580. Epub 2019 Nov 28 |
| 9. | Custódio NB, Costa FDS, Cademartori MG, da Costa VPP, Goettems ML. Effectiveness of Virtual Reality Glasses as a Distraction for Children During Dental Care. Pediatr Dent. 2020 Mar 15;42(2):93-102. PMID: 32276674. |
| 10. | Patnana AK, Chugh VK, Chugh A, Vanga NRV, Kumar P. Effectiveness of zirconia crowns compared with stainless steel crowns in primary posterior teeth rehabilitation: A systematic review and meta-analysis. J Am Dent Assoc. 2022 Feb;153(2):158-166.e5. doi: 10.1016/j.adaj.2021.08.005. PMID: 35086644. |
| 11. | Davidovich E, Shafir S, Shay B, Zini A. Plaque Removal by a Powered Toothbrush Versus a Manual Toothbrush in Children: A Systematic Review and Meta-Analysis. Pediatr Dent. 2020 Jul 15;42(4):280-287. PMID: 32847667 |
| 12. | Monteiro J, Tanday A, Ashley PF, Parekh S, Alamri H. Interventions for increasing acceptance of local anaesthetic in children and adolescents having dental treatment. Cochrane Database Syst Rev. 2020 Feb 27;2(2):CD011024. doi: 10.1002/14651858.CD011024.pub2. PMID: 32104910; PMCID: PMC7045283 |
| 13. | Benson PE, Atwal A, Bazargani F, Parkin N, Thind B. Interventions for promoting the eruption of palatally displaced permanent canine teeth, without the need for surgical exposure, in children aged 9 to 14 years. Cochrane Database Syst Rev. 2021 Dec 30;12(12):CD012851. doi: 10.1002/14651858.CD012851.pub2. PMID: 34967448; PMCID: PMC8717471. |
| 14. | Lai YYL, Zafar S, Leonard HM, Walsh LJ, Downs JA. Oral health education and promotion in special needs children: Systematic review and meta-analysis. Oral Dis. 2022 Jan;28(1):66-75. doi: 10.1111/odi.13731. Epub 2020 Dec 2. PMID: 33215786 |
| 15. | Kashbour W, Gupta P, Worthington HV, Boyers D. Pit and fissure sealants versus fluoride varnishes for preventing dental decay in the permanent teeth of children and adolescents. Cochrane Database Syst Rev. 2020 Nov 4;11:CD003067. doi: 10.1002/14651858.CD003067.pub5. PMID: 33142363. |
| 16. | El Kady DM, Khater AGA, Schwendicke F. Chlorhexidine to improve the survival of ART restorations: A systematic review and meta-analysis. J Dent. 2020 Dec;103:103491. doi: 10.1016/j.jdent.2020.103491. Epub 2020 Sep 30. |
| 17. | Hao S, Ji L, Wang Y. Effect of Honey on Pediatric Radio/Chemotherapy-Induced Oral Mucositis (R/CIOM): A Systematic Review and Meta-Analysis. Evid Based Complement Alternat Med. 2022 Mar 18;2022:6906439. doi: 10.1155/2022/6906439 |
| 18. | Santos GM, Pacheco RL, Bussadori SK, Santos EM, Riera R, de Oliveira Cruz Latorraca C, Mota P, Benavent Caldas Bellotto EF, Martimbianco ALC. Effectiveness and Safety of Ozone Therapy in Dental Caries Treatment: Systematic Review and Meta-analysis. J Evid Based Dent Pract. 2020 Dec;20(4):101472. doi: 10.1016/j.jebdp.2020.101472. Epub 2020 Jul 29. PMID: 33303100 |
| 19. | Chua H, Sardana D, Turner R, Ting G, Ekambaram M. Effectiveness of oral health education methods on oral hygiene in children and adolescents with visual impairment: A systematic review. Int J Paediatr Dent. 2021 Nov;31(6):724-741. doi: 10.1111/ipd.12788. Epub 2021 May 6. PMID: 33730385. |
| 20. | Yu L, Yu X, Li Y, Yang F, Hong J, Qin D, Song G, Hua F. The additional benefit of professional fluoride application for children as an adjunct to regular fluoride toothpaste: a systematic review and meta-analysis. Clin Oral Investig. 2021 Jun;25(6):3409-3419. doi: 10.1007/s00784-021-03909-5. Epub 2021 Mar 29. PMID: 33782769. |
| 21. | Xiang B, Wong HM, Perfecto AP, McGrath CPJ. The application of theory-guided oral health interventions in adolescents: a systematic review and meta-analysis of randomized controlled trials. Psychol Health2021 Jul;36(7):879-894. doi: 10.1080/08870446.2020.1801679. |
| 22. | Gupta A, Sharda S, Nishant, Shafiq N, Kumar A, Goyal A. Topical fluoride-antibacterial agent combined therapy versus topical fluoride monotherapy in preventing dental caries: a systematic review and meta-analysis. Eur Arch Paediatr Dent. 2020 Dec;21(6):629-646. doi: 10.1007/s40368-020-00561-7. Epub 2020 Oct 1. PMID: 33006116. |
| 23. | Ramamurthy P, Rath A, Sidhu P, Fernandes B, Nettem S, Fee PA, Zaror C, Walsh T. Sealants for preventing dental caries in primary teeth. Cochrane Database Syst Rev. 2022 Feb 11;2(2):CD012981. doi: 10.1002/14651858.CD012981.pub2. PMID: 35146744; PMCID: PMC8832104. |
| 24. | Mota KR, da Silva JVF, Borges CD, Leite de Marcelos PGC, Alvares PR, Santos Júnior VED. Effectiveness of the use of xylitol chewing gum in prevention of dental caries: A systematic review. J Indian Soc Pedod Prev Dent. 2021 Apr-Jun;39(2):113-119. doi: 10.4103/JISPPD.JISPPD_330_20. PMID: 34341229. |

**Table S3**. List of studies excluded after full-text analysis and exclusion criteria.

|  | Maulanti T, Nurmala I. A systematic review of oral health educational media innovation for visually impaired children: Which one brings the best impact of change? Spec Care Dentist. 2021;41(4):442-452. doi: 10.1111/scd.12592. PMID: 33825212. | The systematic review did not assess the certainty of evidence through the GRADE approach. |
| --- | --- | --- |
|  | Cunningham, A., McPolin, O., Fallis, R. *et al.* A systematic review of the use of virtual reality or dental smartphone applications as interventions for management of paediatric dental anxiety. BMC Oral Health. 2021; 244  DOI: 10.1186/s12903-021-01602-3 | The systematic review did not assess the certainty of evidence through the GRADE approach. |
|  | Taylor GD, Carr K, Rogers HJ, Vernazza CR. A systematic review of the quality and scope of decision modelling studies in child oral health research. BMC Oral Health. 2021;21(1):318. doi: 10.1186/s12903-021-01680-3. PMID: 34167525; PMCID: PMC8229274. | The systematic review did not assess the certainty of evidence through the GRADE approach. |
|  | Taylor, G. D., Vernazza, C. R., & Abdulmohsen, B. (2019). Success of endodontic management of compromised first permanent molars in children: a systematic review. International Journal of Paediatric Dentistry. doi:10.1111/ipd.12599 | Systematic reviews /meta-analysis of observational studies of exposures related to the outcome (PECO). |
|  | Achmad H, Huldani, Inayah NH, Ramadhany Y. A Systematic Review of Oral Myofunctional Therapy for Future Treatment in Pediatric Obstructive Sleep Apnea (OSA). Systematic Reviews in Pharmacy. 2020; 11(6): 522-528 | The systematic review did not assess the certainty of evidence through the GRADE approach. |
|  | Visconti A, Hayes E, Ealy K, Scarborough DR. A systematic review: The effects of frenotomy on breastfeeding and speech in children with ankyloglossia. Int J Speech Lang Pathol. 2021;23(4):349-358. doi: 10.1080/17549507.2020.1849399. PMID: 33501864. | The systematic review did not assess the certainty of evidence through the GRADE approach. |
|  | Chouchene F, Masmoudi F, Baaziz A, Maatouk F, Ghedira H. Antibiotic Mixtures in Noninstrumental Endodontic Treatment of Primary Teeth with Necrotic Pulps: A Systematic Review. Int J Dent. 2021; 2021:5518599. doi: 10.1155/2021/5518599. PMID: 34135965; PMCID: PMC8175181. | The systematic review did not assess the certainty of evidence through the GRADE approach. |
|  | Menon P A, Shivakumar S, Bhambani G, Singh TP, Khare A, Pathak A. Effectiveness of school-based oral health education in influencing oral health among school children-systematic review and meta-analysis. J Head Neck Physicians Surg 2021;9:100-7 | The systematic review did not assess the certainty of evidence through the GRADE approach. |
|  | Söderling E, Pienihäkkinen K. Effects of xylitol and erythritol consumption on mutans streptococci and the oral microbiota: a systematic review. Acta Odontol Scand. 2020 Nov;78(8):599-608. doi: 10.1080/00016357.2020.1788721. Epub 2020 Jul 7. PMID: 32633595 | The systematic review did not assess the certainty of evidence through the GRADE approach. |
|  | Cardoso M, Coelho A, Lima R, Amaro I, Paula A, Marto CM, Sousa J, Spagnuolo G, Marques Ferreira M, Carrilho E. Efficacy and Patient's Acceptance of Alternative Methods for Caries Removal-a Systematic Review. J Clin Med. 2020 Oct 23;9(11):3407. doi: 10.3390/jcm9113407. PMID: 33114249; PMCID: PMC7690910. | The systematic review did not assess the certainty of evidence through the GRADE approach. |
|  | Nuvvula S, Nunna M, Almaz ME, Mallineni SK. Efficacy of Licorice Lollipops in Reducing Dental Caries in a Paediatric Population: A Systematic Review. Oral Health Prev Dent. 2020;18(1):97-102. doi: 10.3290/j.ohpd.a44138. PMID: 32238980. | The systematic review did not assess the certainty of evidence through the GRADE approach. |
|  | Hamrah MH, Mokhtari S, Hosseini Z, Khosrozadeh M, Hosseini S, Ghafary ES, Hamrah MH, Narges Tavana. Evaluation of the Clinical, Child, and Parental Satisfaction with Zirconia Crowns in Maxillary Primary Incisors: A Systematic Review. Int J Dent. 2021 Jul 5;2021:7877728. doi: 10.1155/2021/7877728. PMID: 34285695; PMCID: PMC8275371. | The systematic review did not assess the certainty of evidence through the GRADE approach. |
|  | Saxena N, Hugar SM, Soneta SP, Joshi RS, Dialani PK, Gokhale N. Evaluation of the Treatment Protocols in the Management of Pulpally Involved Young Permanent Teeth in Children: A Systematic Review and Meta-analysis. Int J Clin Pediatr Dent. 2022;15(Suppl 1):S103-S113. doi: 10.5005/jp-journals-10005-2218. PMID: 35645501; PMCID: PMC9108820. | The systematic review did not assess the certainty of evidence through the GRADE approach. |
|  | Aliakbari E, Gray-Burrows KA, Vinall-Collier KA, Edwebi S, Salaudeen A, Marshman Z, McEachan RRC, Day PF. Facilitators and barriers to home-based toothbrushing practices by parents of young children to reduce tooth decay: a systematic review. Clin Oral Investig. 2021 Jun;25(6):3383-3393. doi: 10.1007/s00784-021-03890-z. Epub 2021 Mar 20. PMID: 33743074; PMCID: PMC8137613. | The systematic review did not assess the certainty of evidence through the GRADE approach. |
|  | Jiang M, Fan Y, Li KY, Lo ECM, Chu CH, Wong MCM. Factors affecting success rate of atraumatic restorative treatment (ART) restorations in children: A systematic review and meta-analysis. J Dent. 2021 Jan;104:103526. doi: 10.1016/j.jdent.2020.103526. Epub 2020 Nov 11. PMID: 33188846. | The systematic review did not assess the certainty of evidence through the GRADE approach. |
|  | Khouqeer NF, Husein D, Chogle S. For pulp treatment in primary teeth, rotary canal instrumentation may be modestly time efficient, but otherwise similar to manual canal instrumentation. J Evid Based Dent Pract. 2021 Mar;21(1):101495. doi: 10.1016/j.jebdp.2020.101495. Epub 2020 Sep 15. PMID: 34051951. | The systematic review did not assess the certainty of evidence through the GRADE approach. |
|  | García-Rosales LE, Sánchez-Molina M,Borré-Ortiz YM. General anesthesia for the dental care of children and people with disabilities. Revista Cubana de Estomatologia, 2022;59(1). | The systematic review did not assess the certainty of evidence through the GRADE approach. |
|  | Tsai C, Raphael S, Agnew C, McDonald G, Irving M. Health promotion interventions to improve oral health of adolescents: A systematic review and meta-analysis. Community Dent Oral Epidemiol. 2020 Dec;48(6):549-560. doi: 10.1111/cdoe.12567. Epub 2020 Aug 7. PMID: 32767825. | The systematic review did not assess the certainty of evidence through the GRADE approach. |
|  | Aliakbari E, Gray-Burrows KA, Vinall-Collier KA, Edwebi S, Marshman Z, McEachan RRC, Day PF. Home-based toothbrushing interventions for parents of young children to reduce dental caries: A systematic review. Int J Paediatr Dent. 2021 Jan;31(1):37-79. doi: 10.1111/ipd.12658. Epub 2020 May 13. PMID: 32333706. | The systematic review did not assess the certainty of evidence through the GRADE approach. |
|  | Verrett C, Wittenberg BM. How can child life specialists help address dental fear and anxiety in children?: A review. Pediatric Dental Journal. 2021; 31(3): 216-223. | The systematic review did not assess the certainty of evidence through the GRADE approach. |
|  | Santamaría RM, Abudrya MH, Gül G, Mourad MS, Gomez GF, Zandona AGF. How to Intervene in the Caries Process: Dentin Caries in Primary Teeth. Caries Res. 2020;54(4):306-323. doi: 10.1159/000508899. Epub 2020 Aug 27. PMID: 32854105. | The systematic review did not assess the certainty of evidence through the GRADE approach. |
|  | Alqahtani ND. Successful treatment modalities for missing lateral incisors- A systematic review. Saudi Dent J. 2021 Sep;33(6):308-315. doi: 10.1016/j.sdentj.2021.07.002. Epub 2021 Jul 14. PMID: 34434032; PMCID: PMC8376679. | The systematic review did not assess the certainty of evidence through the GRADE approach. |
|  | Surendranath P, Krishnappa S, Srinath S. Silver Diamine Fluoride in Preventing Caries: A Review of Current Trends. Int J Clin Pediatr Dent. 2022;15(Suppl 2):S247-S251. doi: 10.5005/jp-journals-10005-2167. PMID: 35645531; PMCID: PMC9108851. | The systematic review did not assess the certainty of evidence through the GRADE approach. |
|  | Loy F, Underwood B, Stevens C. Watch and learn? A systematic review comparing oral health educational videos with written patient information aimed at parents/carers or children. Br Dent J. 2021 Nov 23:1–6. doi: 10.1038/s41415-021-3616-5. Epub ahead of print. PMID: 34815480; PMCID: PMC8609984. | The systematic review did not assess the certainty of evidence through the GRADE approach. |
|  | Alrashdi M, Ardoin J, Liu JA. Zirconia crowns for children: A systematic review. Int J Paediatr Dent. 2022 Jan;32(1):66-81. doi: 10.1111/ipd.12793. Epub 2021 Apr 25. PMID: 33772904. | The systematic review did not assess the certainty of evidence through the GRADE approach. |
|  | Morzycki A, Nickel K, Newton D, Guilfoyle, R. In search of the optimal pain management strategy for children undergoing cleft lip and palate repair: A systematic review and meta-analysis. Cleft Palate-Craniofacial Journal; 2020;57(4). | The systematic review did not assess the certainty of evidence through the GRADE approach. |
|  | Aldrin Joshua A, Keerthi Sasanka L, Jayaraj G, Ganapathy, D. Review on caries preventive effect of fluoride toothpaste. Indian Journal of Forensic Medicine and Toxicology. 2020; 14(4): 5343-5351. | The systematic review did not assess the certainty of evidence through the GRADE approach. |
|  | Mundra LS, Lowe KM, Khechoyan DY. Alveolar Bone Graft Timing in Patients With Cleft Lip & Palate. J Craniofac Surg. 2022 Jan-Feb 01;33(1):206-210. doi: 10.1097/SCS.0000000000007890. PMID: 34967522. | The systematic review did not assess the certainty of evidence through the GRADE approach. |
|  | Pinheiro FHSL, Drummond RJ, Frota CM, Bartzela TN, Dos Santos PB. Comparison of early and conventional autogenous secondary alveolar bone graft in children with cleft lip and palate: A systematic review. Orthod Craniofac Res. 2020 Nov;23(4):385-397. doi: 10.1111/ocr.12394 | The systematic review did not assess the certainty of evidence through the GRADE approach. |
|  | Duarte NT, Rech BO, Martins IG, Franco JB, Ortega KL. Can children be affected by bisphosphonate-related osteonecrosis of the jaw? A systematic review. Int J Oral Maxillofac Surg. 2020 Feb;49(2):183-191. doi: 10.1016/j.ijom.2019.08.004. Epub 2019 Aug 23. PMID: 31447218. | The systematic review did not assess the certainty of evidence through the GRADE approach. |
|  | Lopes-Fatturi A, Wambier L, Rolim TZC, Reis A, de Souza JF. Restorative Techniques for Permanent First Molars Affected by Hypomineralization: A Systematic Review. Pediatr Dent. 2022 Jan 15;44(1):17-24. PMID: 35232531. | The systematic review did not assess the certainty of evidence through the GRADE approach. |
|  | Ismail AF, Tengku Azmi TMA, Malek WMSWA, Mallineni SK. The effect of multisensory-adapted dental environment on children's behavior toward dental treatment: A systematic review. J Indian Soc Pedod Prev Dent. 2021 Jan-Mar;39(1):2-8. doi: 10.4103/jisppd.jisppd_36_21. PMID: 33885380. | The systematic review did not assess the certainty of evidence through the GRADE approach. |
|  | Goswami M, Sangal A, Rahman B, Chawla S. Comparison of the safety and efficacy of dexmedetomidine with midazolam for the management of paediatric dental patients: A systematic review. J Indian Soc Pedod Prev Dent. 2021 Jul-Sep;39(3):233-239. doi: 10.4103/jisppd.jisppd_517_20. | The systematic review did not assess the certainty of evidence through the GRADE approach. |
|  | Arduim ADS, Gonçalves DP, Casagrande L, Lenzi TL. Is lentulospiral the best option for root canal filling of endodontically treated primary teeth? A systematic review and meta-analysis. Eur Arch Paediatr Dent. 2021 Aug;22(4):537-545. doi: 10.1007/s40368-021-00615-4. Epub 2021 Mar 24. PMID: 33763823. | The systematic review did not assess the certainty of evidence through the GRADE approach. |
|  | Garrocho-Rangel A, Esparza-Villalpando V, Pozos-Guillen A. Outcomes of direct pulp capping in vital primary teeth with cariously and non-cariously exposed pulp: A systematic review. Int J Paediatr Dent. 2020 Sep;30(5):536-546. doi: 10.1111/ipd.12633. Epub 2020 Mar 9. PMID: 32078201. | The systematic review did not assess the certainty of evidence through the GRADE approach. |
|  | Garbim JR, Laux CM, Tedesco TK, Braga MM, Raggio DP. Atraumatic restorative treatment restorations performed in different settings: systematic review and meta-analysis. Aust Dent J. 2021 Dec;66(4):430-443. doi: 10.1111/adj.12871. Epub 2021 Sep 2. PMID: 34407233. | The systematic review did not assess the certainty of evidence through the GRADE approach. |
|  | Yu J, Liu S, Zhang X. Can buccal infiltration of articaine replace traditional inferior alveolar nerve block for the treatment of mandibular molars in pediatric patients?: A systematic review and meta-analysis. Med Oral Patol Oral Cir Bucal. 2021 Nov 1;26(6):e754-e761. doi: 10.4317/medoral.24726 | The systematic review did not assess the certainty of evidence through the GRADE approach. |
|  | Boutsiouki C, Frankenberger R, Krämer N. Clinical and radiographic success of (partial) pulpotomy and pulpectomy in primary teeth: A systematic review. Eur J Paediatr Dent. 2021 Dec;22(4):273-285. doi: 10.23804/ejpd.2021.22.04.4. | The systematic review did not assess the certainty of evidence through the GRADE approach. |
|  | Heikel T, Patel S, Ziai K, Shah SJ, Lighthall JG. Botulinum Toxin A in the Management of Pediatric Sialorrhea: A Systematic Review. Ann Otol Rhinol Laryngol. 2022 Feb 18:34894221078365. doi: 10.1177/00034894221078365. Epub ahead of print. PMID: 35176902. | The systematic review did not assess the certainty of evidence through the GRADE approach. |
|  | Mazur M, Ndokaj A, Marasca B, Sfasciotti GL, Marasca R, Bossù M, Ottolenghi L, Polimeni A. Clinical Indications to Germectomy in Pediatric Dentistry: A Systematic Review. Int J Environ Res Public Health. 2022 Jan 10;19(2):740. doi: 10.3390/ijerph19020740. PMID: 35055565; PMCID: PMC8775662. | The systematic review did not assess the certainty of evidence through the GRADE approach. |
|  | Gupta A, Nishant, Sharda S, Kumar A, Goyal A, Gauba K. Comparing the Effectiveness of Topical Fluoride and Povidone Iodine with Topical Fluoride Alone for the Prevention of Dental Caries among Children: A Systematic Review and Meta-analysis. Int J Clin Pediatr Dent. 2020 Sep-Oct;13(5):559-565. doi: 10.5005/jp-journals-10005-1844. PMID: 33623347; PMCID: PMC7887176. | The systematic review did not assess the certainty of evidence through the GRADE approach. |
|  | Bossù M, Iaculli F, Di Giorgio G, Salucci A, Polimeni A, Di Carlo S. Different Pulp Dressing Materials for the Pulpotomy of Primary Teeth: A Systematic Review of the Literature. J Clin Med. 2020 Mar 19;9(3):838. doi: 10.3390/jcm9030838. PMID: 32204501; PMCID: PMC7141304. | The systematic review did not assess the certainty of evidence through the GRADE approach. |
|  | Jabin Z, Vishnupriya V, Agarwal N, Nasim I, Jain M, Sharma A. Effect of 38% silver diamine fluoride on control of dental caries in primary dentition: A Systematic review. J Family Med Prim Care. 2020 Mar 26;9(3):1302-1307. doi: 10.4103/jfmpc.jfmpc_1017_19. | The systematic review did not assess the certainty of evidence through the GRADE approach. |
|  | Amrollahi N, Rastghalam N, Faghihian R. EFFECT OF PRE-COOLING ON PAIN ASSOCIATED WITH DENTAL INJECTIONS IN CHILDREN: A SYSTEMATIC REVIEW. J Evid Based Dent Pract. 2021 Sep;21(3):101588. doi: 10.1016/j.jebdp.2021.101588. Epub 2021 Jun 4. PMID: 34479670. | The systematic review did not assess the certainty of evidence through the GRADE approach. |
|  | Bandyopadhyay A, Kaneshiro K, Camacho M. Effect of myofunctional therapy on children with obstructive sleep apnea: a meta-analysis. Sleep Med. 2020 Nov;75:210-217. doi: 10.1016/j.sleep.2020.08.003. Epub 2020 Aug 10. PMID: 32861058. | The systematic review did not assess the certainty of evidence through the GRADE approach. |
|  | Antonarakis GS, Blanc A, Courvoisier DS, Scolozzi P. Effect of intra-articular corticosteroid injections on pain and mouth opening in juvenile idiopathic arthritis with temporomandibular involvement: A systematic review and meta-analysis. J Craniomaxillofac Surg. 2020 Aug;48(8):772-778. doi: 10.1016/j.jcms.2020.06.010. Epub 2020 Jun 27. PMID: 32680671. | The systematic review did not assess the certainty of evidence through the GRADE approach. |
|  | Paisi M, Plessas A, Pampaka D, Burns L, Witton R. Effect of treating carious teeth on children's and adolescents' anthropometric outcomes: A systematic review of randomised controlled trials. Community Dent Health. 2020 Feb 27;37(1):32-38. doi: 10.1922/CDH_4611Paisi07. PMID: 32031345. | The systematic review did not assess the certainty of evidence through the GRADE approach. |
|  | Weber KR, Wierichs RJ, Meyer-Lueckel H, Flury S. Restoration of teeth affected by molar-incisor hypomineralisation: a systematic review. Swiss Dent J. 2021 Dec 6;131(12):988-997. Epub 2021 Mar 25. PMID: 33764037. | The systematic review did not assess the certainty of evidence through the GRADE approach. |
|  | Faisal MR, Mishu MP, Jahangir F, Younes S, Dogar O, Siddiqi K, Torgerson DJ. The effectiveness of behaviour change interventions delivered by non-dental health workers in promoting children's oral health: A systematic review and meta-analysis. PLoS One. 2022 Jan 11;17(1):e0262118. doi: 10.1371/journal.pone.0262118. PMID: 35015771; PMCID: PMC8751985. | The systematic review did not assess the certainty of evidence through the GRADE approach. |
|  | Jafarzadeh D, Rezapour R, Abbasi T, Tabrizi JS, Zeinolabedini M, Khalili A, Yousefi M. The Effectiveness of Fluoride Varnish and Fissure Sealant in Elementary School Children: A Systematic Review and Meta-Analysis. IRANIAN JOURNAL OF PUBLIC HEALTH. 2022; 51(2); 266-277. | The systematic review did not assess the certainty of evidence through the GRADE approach. |
|  | Anopa Y, Macpherson L, McIntosh E. Systematic Review of Economic Evaluations of Primary Caries Prevention in 2- to 5-Year-Old Preschool Children. Value Health. 2020 Aug;23(8):1109-1118. doi: 10.1016/j.jval.2020.04.1823. Epub 2020 Jul 12. PMID: 32828224. | The systematic review did not assess the certainty of evidence through the GRADE approach. |
|  | Shen A, Bernabé E, Sabbah W. Systematic Review of Intervention Studies Aiming at Reducing Inequality in Dental Caries among Children. Int J Environ Res Public Health. 2021 Feb 1;18(3):1300. doi: 10.3390/ijerph18031300. PMID: 33535581; PMCID: PMC7908536 | The systematic review did not assess the certainty of evidence through the GRADE approach. |
|  | Yarom N, Hovan A, Bossi P, Ariyawardana A, Jensen SB, Gobbo M, Saca-Hazboun H, Kandwal A, Majorana A, Ottaviani G, Pentenero M, Nasr NM, Rouleau T, Lucas AS, Treister NS, Zur E, Ranna V, Vaddi A, Barasch A, Lalla RV, Cheng KKF, Elad S; Mucositis Study Group of the Multinational Association of Supportive Care in Cancer / International Society of Oral Oncology (MASCC/ISOO). Systematic review of natural and miscellaneous agents, for the management of oral mucositis in cancer patients and clinical practice guidelines - part 2: honey, herbal compounds, saliva stimulants, probiotics, and miscellaneous agents. Support Care Cancer. 2020 May;28(5):2457-2472. doi: 10.1007/s00520-019-05256-4. | The systematic review did not assess the certainty of evidence through the GRADE approach. |
|  | Gonsalves CL, Zhu JW, Kim GY, Leveille CF, Kam AJ. Surgical versus conservative management of tongue lacerations in the acute care setting: A systematic review of the literature. Paediatr Child Health. 2021 Aug 3;27(1):32-42. doi: 10.1093/pch/pxab044. PMID: 35273669; PMCID: PMC8900692. | The systematic review did not assess the certainty of evidence through the GRADE approach. |
|  | Mahmoudinezhad Dezfouli SM, Khosravi S, Dezfouli SMM, Khosravi S. Systematic review of the effective factors in pain management in children. PAKISTAN JOURNAL OF MEDICAL & HEALTH SCIENCES. 2020; 14(2): 1236-1243. | The systematic review did not assess the certainty of evidence through the GRADE approach. |
|  | Adobes Martin M, Lipani E, Alvarado Lorenzo A, Bernes Martinez L, Aiuto R, Dioguardi M, Re D, Paglia L, Garcovich D. The effect of maxillary protraction, with or without rapid palatal expansion, on airway dimensions: A systematic review and meta-analysis. Eur J Paediatr Dent. 2020 Dec;21(4):262-270. doi: 10.23804/ejpd.2020.21.04.2. PMID: 33337900. | The systematic review did not assess the certainty of evidence through the GRADE approach. |
|  | Achmad H, Taya. The use of space maintainer in pediatric dentistry: A systematic review. European Journal of Molecular and Clinical Medicine. 2021;8(2): 1532-1545. | The systematic review did not assess the certainty of evidence through the GRADE approach. |
|  | Ajayakumar LP, Chowdhary N, Reddy VR, Chowdhary R. Use of Restorative Full Crowns Made with Zirconia in Children: A Systematic Review. Int J Clin Pediatr Dent. 2020 Sep-Oct;13(5):551-558. doi: 10.5005/jp-journals-10005-1822. PMID: 33623346; PMCID: PMC7887175. | The systematic review did not assess the certainty of evidence through the GRADE approach. |
|  | Wakhloo T, Reddy SG, Sharma SK, Chug A, Dixit A, Thakur K. Silver Diamine Fluoride Versus Atraumatic Restorative Treatment in Pediatric Dental Caries Management: A Systematic Review and Meta-analysis. J Int Soc Prev Community Dent. 2021 Jul 3;11(4):367-375. doi: 10.4103/jispcd.JISPCD_83_21. PMID: 34430496; PMCID: PMC8352060. | The systematic review did not assess the certainty of evidence through the GRADE approach. |
|  | Ortiz-Ruiz AJ, Pérez-Guzmán N, Rubio-Aparicio M, Sánchez-Meca J. Success rate of proximal tooth-coloured direct restorations in primary teeth at 24 months: a meta-analysis. Sci Rep. 2020 Apr 14;10(1):6409. doi: 10.1038/s41598-020-63497-4. PMID: 32286461; PMCID: PMC7156457. | The systematic review did not assess the certainty of evidence through the GRADE approach. |
|  | Tharakan AP, Pawar M, Kale S. Effectiveness of licorice in preventing dental caries in children: A systematic review. J Indian Soc Pedod Prev Dent. 2020 Oct-Dec;38(4):325-331. doi: 10.4103/JISPPD.JISPPD_100_20. PMID: 33402612 | The systematic review did not assess the certainty of evidence through the GRADE approach. |
|  | Goswami M, Sangal A, Rahman B, Chawla S. Comparison of the safety and efficacy of dexmedetomidine with midazolam for the management of paediatric dental patients: A systematic review. J Indian Soc Pedod Prev Dent. 2021 Jul-Sep;39(3):233-239. doi: 10.4103/jisppd.jisppd_517_20. | The systematic review did not assess the certainty of evidence through the GRADE approach. |
|  | Ismail AF, Tengku Azmi TMA, Malek WMSWA, Mallineni SK. The effect of multisensory-adapted dental environment on children's behavior toward dental treatment: A systematic review. J Indian Soc Pedod Prev Dent. 2021 Jan-Mar;39(1):2-8. doi: 10.4103/jisppd.jisppd_36_21. PMID: 33885380 | The systematic review did not assess the certainty of evidence through the GRADE approach. |
|  | Ranzer M, Daniele E, Purnell CA. Perioperative Management of Cleft Lip Repair: A Meta-Analysis and Clinical Practice Guideline. Cleft Palate Craniofac J. 2021 Oct;58(10):1217-1225. doi: 10.1177/1055665620984909. | The systematic review did not assess the certainty of evidence through the GRADE approach. |
|  | Brignardello-Petersen R. Accuracy for predicting caries in preschool-aged children seems to vary across risk assessment tools. J Am Dent Assoc. 2020;151(4):e32. doi: 10.1016/j.adaj.2019.11.003. | The study is not a systematic review. |
|  | Anopa Y, Conway DI. Exploring the cost-effectiveness of child dental caries prevention programmes. Are we comparing apples and oranges? Evid Based Dent. 2020 Mar;21(1):5-7. doi: 10.1038/s41432-020-0085-7. | The study is not a systematic review. |
|  | Timms L, Deery C. Fluoride varnish and dental caries in preschoolers: a systematic review and meta-analysis. Evid Based Dent. 2020 Mar;21(1):18-19. doi: 10.1038/s41432-020-0074-x. | The study is not a systematic review. |
|  | Bakhurji E. Fluoride Varnish Application in Preschoolers Have a Modest Effectiveness in Reducing the Incidence of Dentinal Caries. J Evid Based Dent Pract. 2020 Dec;20(4):101489. doi: 10.1016/j.jebdp.2020.101489. | The study is not a systematic review. |
|  | Hassan MM. Glass Ionomer Cements May Be Used as an Alternative to Composite Resins in Class II (CL II) Restoration of Primary Molars. J Evid Based Dent Pract. 2020 Jun;20(2):101437. doi: 10.1016/j.jebdp.2020.101437. | The study is not a systematic review. |
|  | Quach H. How can children be involved in developing oral health education interventions? Evid Based Dent. 2020 Sep;21(3):104-105. doi: 10.1038/s41432-020-0122-6. | The study is not a systematic review. |
|  | Schmoeckel J, Gorseta K, Splieth CH, Juric H. How to Intervene in the Caries Process: Early Childhood Caries - A Systematic Review. Caries Res. 2020;54(2):102-112. doi: 10.1159/000504335. | The study is not a systematic review. |
|  | Lee JJ, Sarangam M, Feldman KW, Tieder JS. Riga-Fede Disease: A Case of Sublingual Trauma Not Associated With Abuse. Pediatr Emerg Care. 2021 Dec 1;37(12):e1735-e1737. doi: 10.1097/PEC.0000000000001922. | The study is not a systematic review. |
|  | Mathur VP, Gowthaman K, Shrivstava N, Atif M, Tewari N, Rahul M, Bansal K. An Insight into Systematic Review and Meta-Analysis of Nonvital Pulp Therapy for Primary Teeth. Pediatr Dent. 2021 Sep 15;43(5):338-339. | The study is not a systematic review. |
|  | Timms L, Deery C. Do panoramic radiographs offer improved diagnostic accuracy over clinical examination and other radiographic techniques in children? Evid Based Dent. 2021 Jan;22(3):110-111. doi: 10.1038/s41432-021-0194-y. | The study is not a systematic review. |
|  | Miranda-Silva W, Gomes-Silva W, Zadik Y, Yarom N, Al-Azri AR, Hong CHL, Ariyawardana A, Saunders DP, Correa ME, Arany PR, Bowen J, Cheng KKF, Tissing WJE, Bossi P, Elad S; Mucositis Study Group of the Multinational Association of Supportive Care in Cancer / International Society for Oral Oncology (MASCC/ISOO). MASCC/ISOO clinical practice guidelines for the management of mucositis: sub-analysis of current interventions for the management of oral mucositis in pediatric cancer patients. Support Care Cancer. 2021 Jul;29(7):3539-3562. doi: 10.1007/s00520-020-05803-4. | The study is not a systematic review. |
|  | Rigotti E, Bianchini S, Nicoletti L, Monaco S, Carrara E, Opri F, Opri R, Caminiti C, Donà D, Giuffré M, Inserra A, Lancella L, Mugelli A, Piacentini G, Principi N, Tesoro S, Venturini E, Staiano A, Villani A, Sesenna E, Vicini C, Esposito S, On Behalf Of The Peri-Operative Prophylaxis In Neonatal And Paediatric Age Pop-NeoPed Study Group. Antimicrobial Prophylaxis in Neonates and Children Undergoing Dental, Maxillo-Facial or Ear-Nose-Throat (ENT) Surgery: A RAND/UCLA Appropriateness Method Consensus Study. Antibiotics (Basel). 2022 Mar 13;11(3):382. doi: 10.3390/antibiotics11030382. | The study is not a systematic review. |
|  | Messner AH, Walsh J, Rosenfeld RM, Schwartz SR, Ishman SL, Baldassari C, Brietzke SE, Darrow DH, Goldstein N, Levi J, Meyer AK, Parikh S, Simons JP, Wohl DL, Lambie E, Satterfield L. Clinical Consensus Statement: Ankyloglossia in Children. Otolaryngol Head Neck Surg. 2020 May;162(5):597-611. doi: 10.1177/0194599820915457. | The study is not a systematic review. |
|  | Auychai P, Neff A, Pitak-Arnnop P. Tongue-Tie children with a severe Hazelbaker score or difficult breastfeeding greatly benefit from frenotomy or frenuloplasty with/without anaesthesia - First do or do no harm? J Stomatol Oral Maxillofac Surg. 2022 Jun;123(3):e76-e81. doi: 10.1016/j.jormas.2021.09.007. | The study is not a systematic review. |
|  | Gold J. Silver Diamine Fluoride Prevents Caries in Primary Teeth Superior to No Treatment, Placebo, or Fluoride Varnish. J Evid Based Dent Pract. 2020 Mar;20(1):101422. doi: 10.1016/j.jebdp.2020.101422. | The study is not a systematic review. |
|  | Faghihian R, Faghihian E, Kazemi A, Tarrahi MJ, Zakizade M. Impact of motivational interviewing on early childhood caries: A systematic review and meta-analysis. J Am Dent Assoc. 2020 Sep;151(9):650-659. doi: 10.1016/j.adaj.2020.06.003. | Participants older than 18 years. |
|  | Milani AJ, Castilho T, Assaf AV, Antunes LS, Antunes LAA. Impact of traumatic dental injury treatment on the Oral Health-Related Quality of Life of children, adolescents, and their family: Systematic review and meta-analysis. Dent Traumatol. 2021 Dec;37(6):735-748. doi: 10.1111/edt.12697. | Participants older than 18 years. |
|  | Schwendicke F, Walsh T, Lamont T, Al-Yaseen W, Bjørndal L, Clarkson JE, Fontana M, Gomez Rossi J, Göstemeyer G, Levey C, Müller A, Ricketts D, Robertson M, Santamaria RM, Innes NP. Interventions for treating cavitated or dentine carious lesions. Cochrane Database Syst Rev. 2021 Jul 19;7(7):CD013039. doi: 10.1002/14651858.CD013039.pub2. | Participants older than 18 years. |
|  | Schalch TO, Martimbianco ALC, Gonçalves MLL, Motta LJ, Santos EM, Cecatto RB, Bussadori SK, Horliana ACRT. Interventions for Early-Stage Pericoronitis: Systematic Review of Randomized Clinical Trials. Antibiotics (Basel). 2022 Jan 8;11(1):71. doi: 10.3390/antibiotics11010071. | Participants older than 18 years. |
|  | Colvara BC, Faustino-Silva DD, Meyer E, Hugo FN, Celeste RK, Hilgert JB. Motivational interviewing for preventing early childhood caries: A systematic review and meta-analysis. Community Dent Oral Epidemiol. 2021 Feb;49(1):10-16. doi: 10.1111/cdoe.12578. | Participants older than 18 years. |
|  | Cai H, Xi P, Zhong L, Chen J, Liang X. Efficacy of aromatherapy on dental anxiety: A systematic review of randomised and quasi-randomised controlled trials. Oral Dis. 2021 May;27(4):829-847. doi: 10.1111/odi.13346. | Participants older than 18 years. |
|  | Fee PA, Riley P, Worthington HV, Clarkson JE, Boyers D, Beirne PV. Recall intervals for oral health in primary care patients. Cochrane Database Syst Rev. 2020 Oct 14;10(10):CD004346. doi: 10.1002/14651858.CD004346.pub5. | Participants older than 18 years. |
|  | Balian A, Cirio S, Salerno C, Wolf TG, Campus G, Cagetti MG. Is Visual Pedagogy Effective in Improving Cooperation Towards Oral Hygiene and Dental Care in Children with Autism Spectrum Disorder? A Systematic Review and Meta-Analysis. Int J Environ Res Public Health. 2021 Jan 18;18(2):789. doi: 10.3390/ijerph18020789. | Systematic reviews /meta-analysis of observational studies of exposures related to the outcome (PECO). |
|  | Padovano WM, Skolnick GB, Naidoo SD, Snyder-Warwick AK, Patel KB. Long-Term Effects of Nasoalveolar Molding in Patients With Unilateral Cleft Lip and Palate: A Systematic Review and Meta-Analysis. Cleft Palate Craniofac J. 2022 Apr;59(4):462-474. doi: 10.1177/10556656211009702. | Systematic reviews /meta-analysis of observational studies of exposures related to the outcome (PECO). |
|  | Joufi AI, Claiborne DM, Shuman D. Oral Health Education and Promotion Activities by Early Head Start Programs in the United States: A systematic review. J Dent Hyg. 2021 Oct;95(5):14-21. | Systematic reviews /meta-analysis of observational studies of exposures related to the outcome (PECO). |
|  | Sabbagh H, Othman M, Khogeer L, Al-Harbi H, Al Harthi A, Abdulgader Yaseen Abdulgader A. Parental acceptance of silver Diamine fluoride application on primary dentition: a systematic review and meta-analysis. BMC Oral Health. 2020 Aug 20;20(1):227. doi: 10.1186/s12903-020-01195-3. | Systematic reviews /meta-analysis of observational studies of exposures related to the outcome (PECO). |
|  | Orabi N, Flores-Mir C, Elshebiny T, Elkordy S, Palomo JM. Pharyngeal airway dimensional changes after orthodontic treatment with premolar extractions: A systematic review with meta-analysis. Am J Orthod Dentofacial Orthop. 2021 Oct;160(4):503-515.e3. doi: 10.1016/j.ajodo.2021.03.013. | Systematic reviews /meta-analysis of observational studies of exposures related to the outcome (PECO). |
|  | Aminabadi NA, Asl Aminabadi N, Jamali Z, Shirazi S. Primary tooth pulpectomy overfilling by different placement techniques: A systematic review and meta-analysis. J Dent Res Dent Clin Dent Prospects. 2020 Fall;14(4):250-261. doi: 10.34172/joddd.2020.043. | Systematic reviews /meta-analysis of observational studies of exposures related to the outcome (PECO). |
|  | Grewcock RE, Innes NPT, Mossey PA, Robertson MD. Caries in children with and without orofacial clefting: A systematic review and meta-analysis. Oral Dis. 2022 Mar 9. doi: 10.1111/odi.14183. | Systematic reviews /meta-analysis of observational studies of exposures related to the outcome (PECO). |
|  | Schroth RJ, Rothney J, Sturym M, Dabiri D, Dabiri D, Dong CC, Grant CG, Kennedy T, Sihra R. A systematic review to inform the development of a Canadian caries risk assessment tool for use by primary healthcare providers. Int J Paediatr Dent. 2021 Nov;31(6):767-791. doi: 10.1111/ipd.12776. | Systematic reviews /meta-analysis of observational studies of exposures related to the outcome (PECO). |
|  | Silva JA, Paiva SM, Pereira DMT, Maia LC, Primo LG, Fonseca-Gonçalves A. Are Behavior Rating Scales Able to Identify Behavioral Changes in Preschool Children Undergoing a Dental Intervention? A Systematic Review. Pesquisa Brasileira em Odontopediatria e Clínica Integrada. 2020; 20. | Systematic reviews /meta-analysis of observational studies of exposures related to the outcome (PECO). |
|  | Delimont NM, Carlson BN. Prevention of dental caries by grape seed extract supplementation: A systematic review. Nutr Health. 2020 Mar;26(1):43-52. doi: 10.1177/0260106019887890. | Systematic reviews /meta-analysis of in vitro studies. |
|  | Jullien S. Prophylaxis of caries with fluoride for children under five years. BMC Pediatr. 2021 Sep 8;21(Suppl 1):351. doi: 10.1186/s12887-021-02702-3. | Narrative review. |
|  | Burgess D, Lizarondo L, Gardner S. Effectiveness of air polishing as a method of oral prophylaxis in the orthodontic setting: a systematic review protocol. JBI Evid Synth. 2020 Aug;18(8):1774-1780. doi: 10.11124/JBISRIR-D-19-00287. | The study is a protocol. |
|  | Yu L, Yu X, Li Y, Li J, Hua F, Song G. Is it necessary for children to receive professional fluoride in addition to regular fluoride toothpaste? Protocol for a systematic review. BMJ Open. 2020 Sep 21;10(9):e037422. doi: 10.1136/bmjopen-2020-037422. | The study is a protocol. |
|  | Arora A, Khattri S, Ismail NM, Kumbargere Nagraj S, Eachempati P. School dental screening programmes for oral health. Cochrane Database Syst Rev. 2019 Aug 8;8(8):CD012595. doi: 10.1002/14651858.CD012595.pub3. | The study was published in 2019. |
|  | Santana LG, de Campos França E, Flores-Mir C, Abreu LG, Marques LS, Martins-Junior PA. Effects of lip bumper therapy on the mandibular arch dimensions of children and adolescents: A systematic review. Am J Orthod Dentofacial Orthop. 2020 Apr;157(4):454-465.e1. doi: 10.1016/j.ajodo.2019.10.014. PMID: 32241352. | The outcome is orthodontic |
|  | Vidigal MTC, Mesquita CM, de Oliveira MN, de Andrade Vieira W, Blumenberg C, Nascimento GG, Pithon MM, Paranhos LR. Impacts of using orthodontic appliances on the quality of life of children and adolescents: systematic review and meta-analysis. Eur J Orthod. 2022 Feb 24:cjac003. doi: 10.1093/ejo/cjac003. | The outcome is orthodontic |
|  | Al-Moghrabi D, Alkadhimi A, Tsichlaki A, Pandis N, Fleming PS. The influence of mobile applications and social media-based interventions in producing behavior change among orthodontic patients: A systematic review and meta-analysis. Am J Orthod Dentofacial Orthop. 2022 Mar;161(3):338-354. doi: 10.1016/j.ajodo.2021.09.009. | The outcome is orthodontic |
|  | Turner S, Harrison JE, Sharif FN, Owens D, Millett DT. Orthodontic treatment for crowded teeth in children. Cochrane Database Syst Rev. 2021 Dec 31;12(12):CD003453. doi: 10.1002/14651858.CD003453.pub2. | The outcome is orthodontic |
|  | Biočić J, Perić B, Kopić V, Brajdić D, Macan D, Krajačić K. Opportunities of orthodontic-surgical therapy of impacted teeth. Acta Stomatologica Croatica. 2020; 54(1). | The outcome is orthodontic |
|  | Leroy R, Bourgeois J, Verleye L, Carvalho JC, Eloot A, Cauwels R, Declerck D. Are systemic antibiotics indicated in children presenting with an odontogenic abscess in the primary dentition? A systematic review of the literature. Clin Oral Investig. 2021 May;25(5):2537-2544. doi: 10.1007/s00784-021-03862-3. | The systematic review did not include any study. |
|  | Oliveira Filho GR, Castilhos CM, Kriegl JP, Bianchi GN. Oral preanesthetic medication in children - comparison between midazolam alone and in combination with ketamine: a systematic review and meta-analysis. Braz J Anesthesiol. 2021 Aug 16:S0104-0014(21)00315-8. doi: 10.1016/j.bjane.2021.07.026. | The outcome is related to the medical field. |
|  | Tedesco TK, Calvo AFB, Pássaro AL, Araujo MP, Ladewig NM, Scarpini S, Lara JS, Braga MM, Gimenez T, Raggio DP. Nonrestorative treatment of initial caries lesion in primary teeth: a systematic review and network meta-analysis. Acta Odontol Scand. 2021 Jan;80(1):1-8. doi: 10.1080/00016357.2021.1928748. | Network meta-analysis. |
|  | Tedesco TK, Reis TM, Mello-Moura ACV, Silva GSD, Scarpini S, Floriano I, Gimenez T, Mendes FM, Raggio DP. Management of deep caries lesions with or without pulp involvement in primary teeth: a systematic review and network meta-analysis. Braz Oral Res. 2020 Nov 13;35:e004. doi: 10.1590/1807-3107bor-2021.vol35.0004. | Network meta-analysis. |
|  | Manchanda S, Sardana D, Liu P, Lee GH, Li KY, Lo EC, Yiu CK. Topical fluoride to prevent early childhood caries: Systematic review with network meta-analysis. J Dent. 2022 Jan;116:103885. doi: 10.1016/j.jdent.2021.103885. | Network meta-analysis. |
