# Supplementary material for: Pediatric dentistry systematic reviews using the GRADE approach: methodological study
Source: BMC Oral Health. 2024 Jul 13;24:787. doi: 10.1186/s12903-024-04542-w (PMC11245772; doi:10.1186/s12903-024-04542-w)
Supplement: Supplementary file 4 — Supplementary Material 4 [file 12903_2024_4542_MOESM4_ESM.docx]

**Title**

Pediatric dentistry systematic reviews using the GRADE approach: methodological study

**Authors**

Rachel Alvarenga-Brant, Sarah Queiroz Notaro, Cristine Miron Stefani, Graziela De Luca Canto, Alexandre Godinho Pereira, Luciana Póvoa-Santos, Ana Clara Souza-Oliveira, Julya Ribeiro Campos, Carolina Castro Martins-Pfeifer

**Supplementary material**

**Supplementary Table S4**. Data extraction of the certainty of the evidence per outcome.

| Study | Study design of included studies | Intervention/comparison | Outcome | Effect Estimate (95%CI) | Favors intervention /comparison | Certainty |
| --- | --- | --- | --- | --- | --- | --- |
| Manchanda et al., 2020 | RCT | Rotatory x manual instrumentation | Clinical success of endodontic treatment | RR = 1.01 (0.91 to 1.12) | Favors rotatory | Moderate |
| Manchanda et al., 2020 | RCT | Rotatory x manual instrumentation | Radiographic success | RR = 0.97 (0.74 to 1.27) | Favors manual | Moderate |
| Manchanda et al., 2020 | RCT | Rotatory x manual instrumentation | Quality of canal filling | RR = 1.20 (0.99 to 1.46) | Favors rotatory | Low |
| Manchanda et al., 2020 | RCT | Rotatory x manual instrumentation | Pain follow-up 6 h | RD = -0.27 (-0.41 to -0.31) | Favors rotatory | Moderate |
| Manchanda et al., 2020 | RCT | Rotatory x manual instrumentation | Pain follow-up 48h | RD = -0.13 (-0.24 to -0.02) | Favors rotatory | Moderate |
| Manchanda et al., 2020 | RCT | Rotatory x manual instrumentation | Instrumentation time | WMD = -3.48 (-4.35 to -2.62) | Favors rotatory | Moderate |
| Manchanda et al., 2020 | RCT | Rotatory x manual instrumentation | Root canal filling time | WMD = -0.38 (-0.59 to -0.18) | Favors rotatory | Low |
| Manchanda et al., 2020 | RCT | Rotatory x manual instrumentation | Cleaning effectiveness | Narrative synthesis | Rotatory similar to manual | Low |
| Custódio et al., 2020 | RCT | Using virtual reality (VR) eyeglasses during dental treatment X children's behavioral techniques, including distraction, direct observation, tell-show do, voice control, nonverbal communication, positive reinforcement, and descriptive praise techniques or inactive controls when no behavioral management technique is mentioned. | Anxiety during local anesthesia | MD = -3.44 (-8.18 to 1.29) | Favors virtual reality (VR) eyeglasses | Moderate |
| Custódio et al., 2020 | RCT | Using virtual reality (VR) eyeglasses during dental treatment X children's behavioral techniques, including distraction, direct observation, tell-show do, voice control, nonverbal communication, positive reinforcement, and descriptive praise techniques or inactive controls when no behavioral management technique is mentioned. | Anxiety during caries removal | MD = 0.29 (-4.45 to 5.04) | Favors other techniques | Moderate |
| Custódio et al., 2020 | RCT | Using virtual reality (VR) eyeglasses during dental treatment X children's behavioral techniques, including distraction, direct observation, tell-show do, voice control, nonverbal communication, positive reinforcement, and descriptive praise techniques or inactive controls when no behavioral management technique is mentioned. | Anxiety with rubber dam | MD = 0.89 (-4.88 to 6.67) | Favors other techniques | Moderate |
| Custódio et al., 2020 | RCT | Using virtual reality (VR) eyeglasses during dental treatment X children's behavioral techniques, including distraction, direct observation, tell-show do, voice control, nonverbal communication, positive reinforcement, and descriptive praise techniques or inactive controls when no behavioral management technique is mentioned. | Anxiety during restorative treatment (assessed with heart rate) | MD = -0.29 (-5.70 to 5.13) | Favors virtual reality (VR) eyeglasses | Moderate |
| Custódio et al., 2020 | RCT | Using virtual reality (VR) eyeglasses during dental treatment X children's behavioral techniques, including distraction, direct observation, tell-show do, voice control, nonverbal communication, positive reinforcement, and descriptive praise techniques or inactive controls when no behavioral management technique is mentioned. | Anxiety during restorative treatment (assessed with MCDAS) | MD = -5.07 (-5.34 to -4.80) | Favors virtual reality (VR) eyeglasses | High |
| Custódio et al., 2020 | RCT | Using virtual reality (VR) eyeglasses during dental treatment X children's behavioral techniques, including distraction, direct observation, tell-show do, voice control, nonverbal communication, positive reinforcement, and descriptive praise techniques or inactive controls when no behavioral management technique is mentioned. | Behavior with local anesthesia | MD= -0.41 (-0.91 to 0.08) | Favors virtual reality (VR) eyeglasses | High |
| Custódio et al., 2020 | RCT | Using virtual reality (VR) eyeglasses during dental treatment X children's behavioral techniques, including distraction, direct observation, tell-show do, voice control, nonverbal communication, positive reinforcement, and descriptive praise techniques or inactive controls when no behavioral management technique is mentioned. | Behavior with caries removal handpiece FLACC | MD= -0.33 (-0.58 to -0.08) | Favors virtual reality (VR) eyeglasses | High |
| Custódio et al., 2020 | RCT | Using virtual reality (VR) eyeglasses during dental treatment X children's behavioral techniques, including distraction, direct observation, tell-show do, voice control, nonverbal communication, positive reinforcement, and descriptive praise techniques or inactive controls when no behavioral management technique is mentioned. | Behavior with rubber dam | MD= 0.17 (-0.33 to 0.68) | Favors other techniques | High |
| Custódio et al., 2020 | RCT | Using virtual reality (VR) eyeglasses during dental treatment X children's behavioral techniques, including distraction, direct observation, tell-show do, voice control, nonverbal communication, positive reinforcement, and descriptive praise techniques or inactive controls when no behavioral management technique is mentioned. | Pain perception with local anesthesia (assessed with FACES) | MD= 0.01 (-0.36 to 0.39) | Similar effect | High |
| Custódio et al., 2020 | RCT | Using virtual reality (VR) eyeglasses during dental treatment X children's behavioral techniques, including distraction, direct observation, tell-show do, voice control, nonverbal communication, positive reinforcement, and descriptive praise techniques or inactive controls when no behavioral management technique is mentioned. | Pain perception with local anesthesia (assessed with FPS-R) | MD= -0.98 (-2.17 to 0.21) | Favors virtual reality (VR) eyeglasses | High |
| Custódio et al., 2020 | RCT | Using virtual reality (VR) eyeglasses during dental treatment X children's behavioral techniques, including distraction, direct observation, tell-show do, voice control, nonverbal communication, positive reinforcement, and descriptive praise techniques or inactive controls when no behavioral management technique is mentioned. | Pain perception with restorative treatment (assessed with FACES) | MD= -0.70 (-1.23 to -0.16) | Favors VR eyeglasses | High |
| Lam et al., 2020 | RCT | Glass ionomer sealant (GIS)/ resin modified (RMGIS) X no sealant. | Caries prevention and arrest | OR= 0.79 (0.50 to 1.25) | Similar effect | Very low |
| Lam et al., 2020 | RCT | RBS X GIS 6 months | Caries prevention and arrest (6 months) | OR= 3.90 (1.06 to 14.4) | GIS is more effective than RBS at 6 months | Low |
| Lam et al., 2020 | RCT | RBS X GIS 18 months | Caries prevention and arrest (18 months) | OR=1.92 (0.68 to 5.40) | Similar effect at 18 months after placement | Low |
| Lam et al., 2020 | RCT | RBS X F-RBS (fluoride-containing sealant) | Caries prevention and arrest (24 months) | OR= 12.2 (0.65 to 226.97) | Similar effect at 24 months | Low |
| Lam et al., 2020 | RCT | Auto polymerized RBS (AP-RBS) X light-polymerized RBS (LP-RBS) | Caries prevention and arrest | OR= 0.58 (0.13 to 2.55) | Similar effect at 24 months | Low |
| Lam et al., 2020 | RCT | RBS + topical fluoride varnish (TFV) X TFV alone (at 12 & 24 months) | Caries prevention and arrest (24 months) | OR= (0.42 (0.16 to 1.07) | Similar effect | Low |
| Lam et al., 2020 | RCT | RBS + TFV X Resin infiltration (RI) + TFV | Caries prevention and arrest (24 months) | OR= 1.35 (0.46 to 4.00) | Similar effect | Very low |
| Lam et al., 2020 | RCT | RBS (no comparison with other material) - just retention rate | Sealant retention (6 months) | Retention rate: 89.79 (86.14 to 92.97) | No comparison with another group. No need to assess the certainty of the evidence. | Very low |
| Lam et al., 2020 | RCT | RBS (no comparison with other material) - just retention rate | Sealant retention (12 months) | Retention rate: 86.81 (83.62 to 89.70) | No comparison with another group. No need to assess the certainty of the evidence. | Very low |
| Lam et al., 2020 | RCT | RBS (no comparison with other material) - just retention rate | Sealant retention (18 months) | Retention rate: 85.94 (82.13 to 89.38) | No comparison with another group. No need to assess the certainty of the evidence. | Very low |
| Lam et al., 2020 | RCT | GIS (no comparison with other material) - just retention rate | Sealant retention (6 months) | Retention rate: 94.85 (91.92 to 97.19) | No comparison with another group. No need to assess the certainty of the evidence. | Very low |
| Lam et al., 2020 | RCT | GIS (no comparison with other material) - just retention rate | Sealant retention (18 months) | Retention rate: 20.18 (17.91 to 22.54) | No comparison with another group. No need to assess the certainty of the evidence. | Very low |
| Chugh et al., 2020 | RCT | Rotatory versus manual filing | Instrumentation time | MD= 5 (3.05 to 6.94) | Favors rotary | Moderate |
| Chugh et al., 2020 | RCT | Rotatory versus manual filing | Quality of obturation | RR= 0.71 (0.53 to 0.95) | Favors rotary | Moderate |
| Chugh et al., 2020 | RCT | Rotatory versus manual filing | Quality of obturation—root as unit of analysis | RR=1.05 (0.88 to 1.25) | Similar effect | Moderate |
| Chugh et al., 2020 | RCT | Rotatory versus manual filing | Quality of obturation—teeth as unit of analysis | RR= 0.53 (0.39 to 0.72) | Favors rotary | High |
| Chugh et al., 2020 | RCT | Rotatory versus manual filing | Obturation time | MD= 0.43( 0.15 to 0.71) | Favors rotary | Low |
| Kamber et al. (2021) | RCTs and NRSI | Sealant vs. No sealant (follow-up 3-27 months; assessed with visual-tactile) | Development (initiation and progression/regression) of demineralization (wsl), visual-tactile assessment (3-27 months follow-up time) | RR= 0.70 (0.53 to 0.93) | Favors sealant | Very low |
| Kamber et al. (2021) | RCTs and NRSI | Coating with fluoride release vs. Coating without fluoride release (follow-up 1-24 months; assessed with visual-tactile) | Development (initiation and progression/regression) of demineralization (wsl), visual-tactile assessment (1-24 months follow-up time) | RR= 0.84 (0.69 to 1.02) | Favors fluoride release | Very low |
| Kamber et al. (2021) | RCTs and NRSI | Glass ionomer cement X resin adhesive | Development (initiation and progression/regression) of demineralization (WSL), visual-tactile assessment (12-24 months follow-up time) | RR= 0.72 (0.34 to 1.54) | No significant difference |  |
| Taneja et al. (2020) | RCTs | Articaine effective X lidocaine | Articaine versus lignocaine in pediatric dental procedures (VAS score) | MD= -0.20(-0.29 to -0.10) | Favors articaine | High |
| Taneja et al. (2020) | RCTs | Articaine effective X lidocaine | Articaine versus lignocaine in pediatric dental procedures (FPS score | MD= -0.46(-0.8 to -0.12) | Favors articaine | High |
| Martins et al., 2020 | RCT | NAP (natural antimicrobial derived from phenolic compounds) X SA (synthetic antimicrobial) | Plaque index (PI) | Analysis mango: MD= 0.822 (0.514 to 1.131) / Analysis neem: 0.788 (0.403 to 1.169) | Favors synthetic antimicrobial (SA). Patients who used naps presented a greater mean of biofilm when compared to patients who used CHX and 'neem.' | Very low |
| Martins et al., 2020 | RCT | NAP (natural antimicrobial derived from phenolic compounds) X SA (synthetic antimicrobial) | Mean according to the period of naps administration and biofilm index evaluation ≤15 days (analysis' other naps') | MD= 0.221 (0.074 to 0.367) | Favors synthetic antimicrobial (AS) | Very low |
| Martins et al., 2020 | RCT | NAP (natural antimicrobial derived from phenolic compounds) X SA (synthetic antimicrobial) | Mean according to the period of naps administration and biofilm index evaluation > 15 days (analysis 'mango') | MD= 0.372 (0.110 to 0.635) | Favors synthetic antimicrobial (AS) | Low |
| Martins et al., 2020 | RCT | NAP (natural antimicrobial derived from phenolic compounds) X SA (synthetic antimicrobial) | Mean according to the period of naps administration and biofilm index evaluation > 15 days (analysis 'neem') | MD= 0.221 (0.074 to 0.367) | Favors synthetic antimicrobial (AS) | Low |
| Martins et al., 2020 | RCT | NAP (natural antimicrobial derived from phenolic compounds) X SA (synthetic antimicrobial) | Mean according to the period of naps administration and biofilm index evaluation Overall (analysis 'mango' and 'neem') | MD= Analysis mango: 0.257 (0.129 to 0.385) / Analysis neem: 0.243 (0.112 to 0.374) | Favors synthetic antimicrobial (AS) | Low |
| Martins et al., 2020 | RCT | NAP (natural antimicrobial derived from phenolic compounds) X SA (synthetic antimicrobial) | Mean of microorganisms count -Total mos (analysis 'oil pulling') | SMD= 0.012 (-0.865 to 0.888) | Similar effect | Low |
| Martins et al., 2020 | RCT | NAP (natural antimicrobial derived from phenolic compounds) X SA (synthetic antimicrobial) | Mean of microorganisms count- S. Mutans (analysis 'Triphala' and 'garlic') | SMD= Analysis Triphala: 0.115 (-0.258 to 0.487) / Analysis garlic: 0.109 (-0.264 to 0.481) | Similar effect | Moderate |
| Martins et al., 2020 | RCT | NAP (natural antimicrobial derived from phenolic compounds) X SA (synthetic antimicrobial) | Mean of microorganisms count - Streptococcus spp. (analysis 'Triphala' and 'garlic') | SMD= 0.430 (-0.293 to 1.152) | Favors synthetic antimicrobial (AS) | Very low |
| Martins et al., 2020 | RCT | NAP (natural antimicrobial derived from phenolic compounds) X SA (synthetic antimicrobial) | Mean of microorganisms count. - Overall (analysis of 'Triphala' and 'garlic') | SMD= Analysis Triphala: 0.160 (-0.150 to 0.470) Analysis garlic: 0.156 (-0.154 to 0.465) | Favors synthetic antimicrobial (AS) | Moderate |
| Monteiro et al., 2020 | RCT | Audiovisual distraction X conventional treatment | Self‐ or observational assessment of intraoperative distress/pain/acceptance of treatment during provision of LA:pain‐related behavior during LA | RR= 0.13 (0.03 to 0.50) | Favors audiovisual distraction | Very low |
| Monteiro et al., 2020 | RCT | Pre‐cooling of the injection site X conventional treatment | Pain | Narrative synthesis | The evidence is uncertain | Very low |
| Monteiro et al., 2020 | RCT | The wand X traditional local anesthetic | Pain-related behavior (muscle tension, crying, verbal protest, resistance, and body movement) | Narrative synthesis | Four studies reported a benefit in using the wand, while the remaining studies' results suggested no difference between the two methods of delivering local anesthesia | Very low |
| Monteiro et al., 2020 | RCT | The wand x sleeper one | Pain-related behavior (muscle tension, crying, verbal protest, resistance, and body movement) | MD: 0.06 (0.01 to 0.11) | The evidence is uncertain | Very low |
| Monteiro et al., 2020 | RCT | Camouflage syringe x conventional syringe | Pain-related behavior (muscle tension, crying, verbal protest, resistance, and body movement) | RR 0.02 (0.00 to 0.37) | The evidence is uncertain | Very low |
| Monteiro et al., 2020 | RCT | Electrical counter‐stimulation X no stimulation | Pain | MD: -1.34 (- 2.35 to -0.33) | The evidence is uncertain | Very low |
| Monteiro et al., 2020 | RCT | Counter stimulation, no distraction x conventional treatment | Distraction distress/pain | Narrative synthesis | The evidence is uncertain | Very low |
| Monteiro et al., 2020 | RCT | Video modeling acclimatization x oral hygiene video | Anxiety/pain during local anesthesia | MD: -37.16 (- 50.94 to -23.38) | Favors video modeling X oral hygiene video | Low |
| Monteiro et al., 2020 | RCT | Video modeling acclimatization for local anesthesia x acclimatization in clinic | Acceptance | MD: 0.01 (-0.33 to 0.35) | The evidence is uncertain | Very low |
| Monteiro et al., 2020 | RCT | Hypnosis X conventional treatment | Pain | MD= 1.79 (3.01 to 0.57 ) | The evidence is uncertain | Very low |
| Elkady et al., 2020 | RCT | Chlorhexidine (CHX) X no CHX | Survival of restorations after one year assessed with ART criteria | OR= 0.79 (0.26 to 2.40) | Similar effect | Low |
| Kashbour et al.,2020 | RCT | Resin‐based fissure sealant X fluoride varnish for preventing dental caries | Dentine caries in permanent molars (yes/no) Follow-up: 2–3 years | OR= 0.67 (0.37 - 1.19) | Favors resin sealant | Very low |
| Kashbour et al.,2020 | RCT | Glass ionomer fissure sealant or resin‐modified glass ionomer fissure sealant X fluoride varnish for preventing dental caries | Dentine caries in permanent molars Follow-up: 1, 2, and 3 years | There is no evidence of a difference between interventions in caries after 1, 2, and 3 years. |  | Very low |
| Kashbour et al.,2020 | RCT | Resin‐based fissure sealant plus fluoride varnish X fluoride varnish alone for preventing dental caries | Dentine caries in permanent molars Follow-up: 2 years | OR= 0.30 (0.17 - 0.55) | Favors sealant + varnish | Very low |
| Benson et al., 2021 | RCT | Primary canine extraction X no extraction | Eruption of palatally displaced canine (PDC) at 12 months (Assessed by clinical observation) | RR= 2.87 (0.90 to 9.23) | Similar effect | Very low |
| Benson et al., 2021 | RCT | Primary canine extraction X no extraction | Referral for surgical exposure of PDC at 12 months | RR= 0.61 (0.29 to 1.28) | Similar effect | Very low |
| Benson et al., 2021 | RCT | Double primary (canine and first molar) extractions X single primary (canine) tooth extraction | Eruption of PDC at mean 18 months (MITT analysis) | RR= 0.68 (0.35 to 1.31) | Favors single extraction | Very low |
| Benson et al., 2021 | RCT | Double primary (canine and first molar) extractions X single primary (canine) tooth extraction | Referred for surgical exposure of the unerupted PDC by a maximum of 48 months (mitt analysis) | RR= 0.31 (0.06 to 1.45) | Favors double extraction | Very low |
| Lai et al., 2020 | RCT | Fluoride X no fluoride | Decayed Missing and Filled Teeth index (DMFT) follow-up: 3 years | MD= 0.96 (1.93 to 0.01) | The evidence suggests that fluoride results in a large reduction in the Decayed Missing and Filled Teeth index. | Low |
| Lai et al., 2020 | RCT | Fluoride X no fluoride | Decayed Missing and Filled Surfaces- increase per 100 surfaces at risk (DMFS/DMFS- increase per 100 surfaces at risk ) follow-up: 3 years. | MD= 0.45 (1.77 to 0.87) | The evidence suggests that fluoride slightly reduces Decayed Missing and Filled Surfaces-increment per 100 surfaces at risk. | Low |
| Lai et al., 2020 | RCT | Fluoride X no fluoride | Debris index- Simplified Oral Hygiene Index (DI-S) follow-up: 2 years | MD= 0.59 (1.59 to 0.41) | Fluoride may reduce/have little to no effect on the Debris index (Simplified Oral Hygiene index), but the evidence is very uncertain. | Very low |
| Lai et al., 2020 | RCT | Fluoride X no fluoride | Calculus index -Simplified Oral hygiene index (CI-S) follow-up: 3 years | MD= 0.31 (0.13 to 0.49) | The evidence suggests that fluoride results in little to no difference in the Calculus Index (Simplified Oral Hygiene Index). | Low |
| Lai et al., 2020 | RCT | Fluoride X no fluoride | Gingival index- (Silness & Loe) follow-up: 3 years | MD= 0.09 (0.34 to 0.52) | The evidence suggests that fluoride results in little to no difference in gingival index | Low |
| Lai et al., 2020 | RCT | Chlorhexidine dentifrice X placebo | Plaque index- pooled follow-up: range 14 days to 21 days | SMD= 1.08 (1.49 to 0.67) | The pooled evidence suggests that chlorhexidine dentifrice results in a large reduction in plaque index. | Low |
| Lai et al., 2020 | RCT | Chlorhexidine dentifrice X placebo | Gingival index- (Silness & Loe) follow-up: range 14 days to 21 days | MD= 0.24 (1.21 to 0.73) | The evidence suggests that chlorhexidine dentifrice results in little to no difference in gingival index | Low |
| Lai et al., 2020 | RCT | Modified toothbrushes X Manual toothbrushes | Plaque index- (Quigley Hein 1962) follow-up: mean 7 days | MD= 0.38 (0.86 to 0.1) | Use of modified toothbrushes probably results in a slight reduction in plaque index | Moderate |
| Lai et al., 2020 | RCT | Electric toothbrushes X manual toothbrush | Plaque index- Pooled follow-up: range 7 days to 28 days | SMD= 0.84 (1.8 to 0.12) | Electric toothbrushes may reduce/have little to no effect on plaque index, but the evidence is very uncertain | Very low |
| Lai et al., 2020 | RCT | Electric toothbrushes X Manual toothbrush | Gingival bleeding index- (Muhlemann 1971) follow-up: mean 28 days | MD= 0.2 (5.78 to 5.38) | Electric toothbrushes may reduce/have little to no effect on the gingival bleeding index, but the evidence is very uncertain. | Very low |
| Lai et al., 2020 | RCT | Talk and model oral hygiene instruction (OHI) X Video (OHI) | Plaque index- (Silness & Loe) follow-up: mean 14 days | MD= 0.27 (0.29 to 0.25) | The evidence suggests OHI using talk and tooth model results in a slight reduction in plaque index | Low |
| Lai et al., 2020 | RCT | Talk and model oral hygiene instruction (OHI) X Video (OHI) | Plaque index- (Silness & Loe) follow-up: mean 42 days | MD= 0.05 (0.28 to 0,18) | Oral hygiene instruction using talk and tooth model may result in a slight reduction in plaque index | Low |
| Lai et al., 2020 | RCT | Talk and model oral hygiene instruction (OHI) X Video (OHI) | Gingival index- (Silness & Loe) follow-up: mean 14 days | MD= 0.25 (0.26 to 0.24) | The evidence suggests OHI using talk and tooth model results in a slight reduction in the gingival index | Low |
| Lai et al., 2020 | RCT | Talk and model oral hygiene instruction (OHI) X Video (OHI) | Gingival index-(Silness & Loe) follow-up: mean 42 days | MD= 0.22 (0.44 to 0) | The evidence suggests OHI using talk and tooth model results in a slight reduction in the gingival index | Low |
| Lai et al., 2020 | RCT | Toothbrushing instruction/OHE X OHE only | Plaque index- (Kobayashi & Ash) follow-up: mean 35 days | The intervention group (n=17) and control group (n=9) data consisted of group means (and adjusted means) but no standard deviations. Findings: Toothbrush instruction + OHI > OHI only p<0.025 ANCOVA F statistic =6.38; df=1/23). | Narrative: Favors Toothbrush instruction + OHI > OHI | Very low |
| Lai et al., 2020 | RCT | Video modelling X control video | Plaque index- modified Podshadley & Haley (used 4 instead of 6 teeth) follow-up: mean 21 days | MD= 0.53 (1.48 to 0.42) | Video modeling may reduce/have little to no effect on plaque index, but the evidence is very uncertain. | Very low |
| Lai et al., 2020 | RCT | Video modelling X control video | Plaque index- modified Podshadley & Haley (used 4 instead of 6 teeth) follow-up: mean 42 days | MD= 0.82 (1.83 to 0.19) | Video modeling may reduce/have little to no effect on plaque index, but the evidence is very uncertain. | Very low |
| Hao et al., 2022 | RCT | Honey group X control group | Recovery Duration of radio/chemotherapy-induced oral mucositis (R/CIOM) | MD= -5.10 (-9.60 to 0.61) | Favors Honey group | Low |
| Hao et al., 2022 | RCT | Honey group X control group | Occurrence of all Grades of R radio/chemotherapy-induced oral mucositis (R/CIOM) | RR= 0.19 (0.12 to 0.30) | Favors Honey group | Moderate |
| Hao et al., 2022 | RCT | Honey group X control group | Occurrence of grades III and IV of radio/chemotherapy-induced oral mucositis (R/CIOM) | RR= 0.18 (0.08 to 0.41) | Favors Honey group | Moderate |
| Chua et al., 2021 | RCT | Braille X audio-tactile (oral hygiene education) | Oral hygiene (plaque, calculus, oral hygiene, gingival health, caries, and oral knowledge) | narrative synthesis | Uncertain evidence | Very low |
| Chua et al., 2022 | RCT | Audio X Braille (oral hygiene education) | Oral hygiene (plaque, calculus, oral hygiene, gingival health, caries, and oral knowledge) | narrative synthesis | Uncertain evidence | Very low |
| Chua et al., 2023 | RCT | Audio-tactile X audio-tactile-Braille (oral hygiene education) | Oral hygiene (plaque, calculus, oral hygiene, gingival health, caries, and oral knowledge) | narrative synthesis | Uncertain evidence | Very low |
| Chua et al., 2024 | RCT | Audio-Braille X audio-tactile (oral hygiene education) | Oral hygiene (plaque, calculus, oral hygiene, gingival health, caries, and oral knowledge) | Narrative synthesis | Uncertain evidence | Very low |
| Xiang et al., 2020 | RCT | Theory-guided interventions X conventional education sessions | Oral health behaviors | Narrative synthesis (different outcome measures) | Conflicting results | Moderate |
| Xiang et al., 2020 | RCT | Theory-guided interventions X conventional education sessions | Gingival health | Narrative synthesis (different outcome measures) | Conflicting results | Low |
| Xiang et al., 2020 | RCT | Theory-guided interventions X conventional education sessions | Oral hygiene status | Short term: MD: –5.94 (–16.39 to 4.51); Long term: MD: -0.25 (-0.46 to -0.04) | Short-term: no preferences /Long-term: Favors theory-guided intervention | Low |
| Yu et al., 2021 | RCT | Regular fluoride toothpaste (RFT) + fluoride varnish (FV) X RFT alone for preventing caries in children / ( FV, fluoride varnish; RFT, regular fluoride toothpaste) | Incidence of caries | RR= 0.91 (0.80 to 1.05) | Favors RFT + FV | Moderate |
| Yu et al., 2021 | RCT | RFT + FV X RFT alone for preventing caries in children / ( FV, fluoride varnish; RFT, regular fluoride toothpaste) | Changes in the prevalence of caries | MD=-0.17 (-0.60 to 0.26) | Favors RFT + FV | Low |
| Yu et al., 2021 | RCT | RFT + FV X RFT alone for preventing caries in children / ( FV, fluoride varnish; RFT, regular fluoride toothpaste) | D(m/e)fs increment | RR= 0.89 (0.78 to 1.01) | Similar effect | Moderate |
| Gupta et al., 2020 | RCT | Combined therapy X TF monotherapy | Dental caries | SMD= -0.12 (-0.2, -0.04 ) | Favors combined therapy | Low |
| Gupta et al., 2020 | RCT | Combined therapy X TF monotherapy | S. Mutans | SMD= -0.11 = (-0.33 , 0.1) | No significant difference | Very low |
| Ramamurthy et al. (2022) | RCT | Fluoride‐releasing resin‐based sealants X no sealants | Development of ≥ 1 new carious lesion (caries incidence) Follow‐up: 12 months | BB OR= 1.21 (0.37 to 3.94) (Becker Balagtas odds ratio) | Favors no sealant | Low |
| Ramamurthy et al. (2022) | RCT | Fluoride‐releasing resin‐based sealants X no sealants | Development of ≥ 1 new carious lesion (caries incidence) Follow‐up: 24 months | BB OR 0.76 (0.41 to 1.42) (Becker Balagtas odds ratio) | Favors resin-based sealant | Low |
| Ramamurthy et al. (2022) | RCT | Glass ionomer‐based sealants X no sealants | Development of at ≥ 1 new carious lesion (caries incidence). Follow‐up: 12–30 months | OR= 0.97 (0.63 to 1.49) | Favors glass ionomer sealant | Low |
| Ramamurthy et al. (2022) | RCT | Glass ionomer‐based sealants X resin‐based sealants | Development of ≥ 1 new carious lesion (caries incidence) Follow‐up: 6–24 months | No studies reported for this outcome | Due to the data collection, analysis, and reporting methods, we could not provide any quantitative estimates for this comparison. | Very low |
| Ramamurthy et al. (2022) | RCT | Glass ionomer‐based sealants X (fluoride‐releasing) resin‐based sealants | Sealant retention Complete or partial retention of sealant Follow‐up: 24 months | BB OR= 0.20 (0.11 to 0.36) (Becker Balagtas odds ratio) | Favors resin-based sealant | Very low |
| Ramamurthy et al. (2022) | RCT | Glass ionomer‐based sealants X (fluoride‐releasing) resin‐based sealants | Adverse event | Narrative synthesis | 1 study reported adverse events as some discomfort, such as nausea among some children. 1 child reported feeling uncomfortable and experienced a strong gag reflex following the application of the glass ionomer-based sealant, while 8 children reported feeling uncomfortable after the fluoride resin-based applications. | Low |
| Ramamurthy et al. (2022) | RCT | Fluoride‐releasing resin‐based sealants X resin‐based sealants | Development of ≥ 1 new carious lesion (caries incidence). Follow-up: 6–24 months | Narrative synthesis | Due to the methods of data collection, analysis, and reporting, we were unable to provide any quantitative estimates for this comparison. | Low |
| Ramamurthy et al. (2022) | RCT | Fluoride‐releasing resin‐based sealants X resin‐based sealants | Sealant retention: Complete or partial retention of sealant. Follow-up: 6–24 months | Narrative synthesis | Due to the different sealant materials evaluated, data reporting (split-mouth studies reported as parallel-group studies), and the very low number of lost sealants, we could not pool these data in a meta-analysis. | Very low |
| Ramamurthy et al. (2022) | RCT | Flowable resin composite X resin‐based sealants | Sealant retention: Complete or partial retention of sealant. Follow-up: 12 months | Narrative synthesis | All sealants were retained or partially retained in both groups. | Low |
| Ramamurthy et al. (2022) | RCT | Autopolymerised sealant X light polymerized sealant | Development of ≥ 1 new carious lesion (caries incidence) Follow‐up: 24–36 months | OR= 0.58 (0.15 to 2.19) | Favors auto-polymerised sealant | Very low |
| Ramamurthy et al. (2022) | RCT | Autopolymerised sealant X light polymerized sealant | Sealant retention: Complete or partial retention of sealant Follow‐up: 24–36 months | OR= 0.68 (0.33 to 1.44) | Favors light polymerized sealant | Very low |
| Mota et al. (2021) | RCT | Chewing gum containing only xylitol X prevention strategies or placebo | Caries prevention | Narrative synthesis | Narrative synthesis Favors xylitol but is very low | Very low |
| Smolarek et al. 2020 | RCT | Computerized X conventional anesthesia | Patient‐related outcome | SMD= - 0.76 (-1.29 to 0.23 ) | Favors computerized | Low |
| Smolarek et al. 2020 | RCT | Computerized X conventional anesthesia | Disruptive behavior (dichotomous data) | RR= 0.81 (0.62 to 1.06) | Similar effect | Very low |
| Smolarek et al. 2020 | RCT | Computerized X conventional anesthesia | Disruptive behaviour(continuous data) | SMD= -0.26 (-0.68 to 0.16) | Similar effect | Very low |
| Patnana et al., 2022 | RCTS | Primary posterior teeth rehabilitation using zirconia crowns (zcs) X primary posterior teeth rehabilitation using stainless steel crowns ( sscs) | Clinical failures | RR= 0.48 (0.15 to 1.52) | Favors primary posterior teeth rehabilitation using ZC | Low |
| Patnana et al., 2022 | RCTS | Primary posterior teeth rehabilitation using zcs X primary posterior teeth rehabilitation using sscs | Gingival Health | MD= -0.32 (-0.42 to -023) | Favors primary posterior teeth rehabilitation using ZC | Moderate |
| Lai et al., 2020 | NRCT | Chlorhexidine dentifrice X placebo | Plaque index -pooled - follow-up: range 10 days to 30 days | SMD= 1.43 (2.08 to 0.77) | Chlorhexidine dentifrice may reduce/have little to no effect on plaque index (CHX vs placebo; no fluoride), but the evidence is very uncertain. | Very low |
| Lai et al., 2020 | NRCT | Chlorhexidine dentifrice X placebo | Plaque index- (Silness & Loe PI) Scale from 0 to 3 follow-up: range 45 days to 60 days. | MD= 0.61 (0.79 to 0.43) | Chlorhexidine dentifrice may reduce/have little to no effect on plaque index- Silness and Loe in the medium term, but the evidence is very uncertain. | Very low |
| Lai et al., 2020 | NRCT | Chlorhexidine dentifrice X placebo | Gingival index- (Loe & Silness GI) follow-up: range 21 days to 30 days | MD= 0.22 (0.31 to 0.13) | Chlorhexidine dentifrice may reduce/have little to no effect on the gingival index (Loe and Silness), but the evidence is very uncertain. | Very low |
| Lai et al., 2020 | NRCT | Chlorhexidine dentifrice X placebo | Gingival index- (Loe & Silness) follow-up: range 45 days to 60 days | MD= 0.47 (1.08 to 0.14) | Chlorhexidine dentifrice may reduce/have little to no effect on the gingival index (Loe and Silness), but the evidence is very uncertain. | Very low |
| Lai et al., 2020 | NRCT | Chlorhexidine dentifrice X placebo | Gingival bleeding index- (Ainamo & Bay)- follow-up: mean 10 days | Narrative synthesis | The evidence about the effect of chlorhexidine dentifrice on the gingival bleeding index (Ainamo & Bay) (CHX dentifrice without fluoride vs. Placebo dentifrice) is very uncertain. | Very low |
| Lai et al., 2020 | NRCT | Yoga and oral health education (OHE) X OHE | Plaque index- (Silness & Loe) follow-up: mean 30 days | MD= 0.03 (0.13 to 0.07) | The evidence about the effect of yoga and OHE on plaque index is very uncertain (Silness & Loe). | Very low |
| Lai et al., 2020 | NRCT | Yoga and oral health education (OHE) X OHE | Plaque index- (Silness & Loe) follow-up: mean 60 days | MD= 0.18 (0.34 to 0.02) | Yoga and OHE may reduce/have little to no effect on plaque index (Silness & Loe), but the evidence is very uncertain. | Very low |
| Lai et al., 2020 | NRCT | Yoga and oral health education (OHE) X OHE | Plaque index- (Silness & Loe PI) follow-up: mean 180 days | MD= 0.39 (0.56 to 0.22) | Plaque index- (Silness & Loe) follow-up: mean 180 days | Very low |
| Lai et al., 2020 | NRCT | Yoga and oral health education (OHE) X OHE | Gingival index- (Loe & Silness GI) follow-up: mean 30 days | MD= 0.08 (0.17 to 0.01) | The evidence about the effect of yoga and OHE on the gingival index (Loe & Silness) is very uncertain. | Very low |
| Lai et al., 2020 | NRCT | Yoga and oral health education (OHE) X OHE | Gingival index- (Loe & Silness GI) follow-up: mean 60 days | MD= 0.16 (0.27 to 0.05) | Yoga and OHE may reduce/have little to no effect on the gingival index (Silness & Loe), but the evidence is very uncertain. | Very low |
| Lai et al., 2020 | NRCT | Yoga and oral health education (OHE) X OHE | Gingival index- (Loe & Silness GI) follow-up: mean 180 days | MD= 0.4 (0.51 to 0.29) | Yoga and OHE may reduce/have little to no effect on the gingival index (Silness & Loe), but the evidence is very uncertain. | Very low |
